# Bifidobacterial β-Galactosidase-Mediated Production of Galacto-Oligosaccharides: Structural and Preliminary Functional Assessments

**DOI:** 10.3389/fmicb.2021.750635

**Published:** 2021-10-28

**Authors:** Valentina Ambrogi, Francesca Bottacini, John Mac Sharry, Justin van Breen, Ellen O’Keeffe, Dan Walsh, Barry Schoemaker, Linqiu Cao, Bas Kuipers, Cordula Lindner, Maria Luisa Jimeno, Elisa G. Doyagüez, Oswaldo Hernandez-Hernandez, F. Javier Moreno, Margriet Schoterman, Douwe van Sinderen

**Affiliations:** ^1^APC Microbiome Ireland, University College Cork, Cork, Ireland; ^2^School of Microbiology, University College Cork, Cork, Ireland; ^3^Department of Biological Sciences, Munster Technological University, Cork, Ireland; ^4^School of Medicine, University College Cork, Cork, Ireland; ^5^FrieslandCampina, Amersfoort, Netherlands; ^6^Centro de Química Orgánica “Lora Tamayo” (CSIC), Madrid, Spain; ^7^Instituto de Investigación en Ciencias de la Alimentación, CIAL (CSIC-UAM), Universidad Autónoma de Madrid, Madrid, Spain

**Keywords:** *Bifidobacterium*, prebiotics, gut microbiota, microbiome, bifidogenic, infant, oligosaccharides

## Abstract

In the current study the ability of four previously characterized bifidobacterial β-galactosidases (designated here as BgaA, BgaC, BgaD, and BgaE) to produce galacto-oligosaccharides (GOS) was optimized. Of these enzymes, BgaA and BgaE were found to be promising candidates for GOS production (and the corresponding GOS mixtures were called GOS-A and GOS-E, respectively) with a GOS concentration of 19.0 and 40.3% (of the initial lactose), respectively. GOS-A and GOS-E were partially purified and structurally characterized. NMR analysis revealed that the predominant (non-lactose) disaccharide was allo-lactose in both purified GOS preparations. The predominant trisaccharide in GOS-A and GOS-E was shown to be 3′-galactosyllactose, with lower levels of 6′-galactosyllactose and 4′-galactosyllactose. These three oligosaccharides have also been reported to occur in human milk. Purified GOS-A and GOS-E were shown to be able to support bifidobacterial growth similar to a commercially available GOS. In addition, GOS-E and the commercially available GOS were shown to be capable of reducing *Escherichia coli* adhesion to a C2BBe1 cell line. Both *in vitro* bifidogenic activity and reduced *E. coli* adhesion support the prebiotic potential of GOS-E and GOS-A.

## Introduction

Bifidobacteria are common gut commensals that are particularly abundant in the intestinal microbiota of naturally delivered, full-term and breast-fed infants (Turroni et al., 2012; Moossavi et al., 2019). Their presence in the human gut, especially in the infant gut, has been associated with various beneficial host effects, such as prevention of allergy, brain development, protection against infection (Fanning et al., 2012), anti-inflammatory activity (Limdi et al., 2006; Kekkonen et al., 2008; Wu et al., 2021), production of Short Chain Fatty Acids (SCFAs) and promoting health in later life.

The composition of the infant gut microbiota is the result of colonization, development and maturation of microbial communities in the intestinal tract of newborns. Several factors have been found or suggested to influence gut colonization by bifidobacteria, among which are birth mode (caesarian vs. vaginal delivery), environment, gestational age (pre-term vs. full-term), feeding type (breast feeding vs. formula feeding), secretor status of the mother (Lewis et al., 2015) and dietary habits of the mother (Penders et al., 2006; Milani et al., 2017; Ahern et al., 2019). In relation to the feeding type, it has been established that breast feeding, when compared to formula feeding, allows a more rapid colonization of the neonatal gut by bifidobacteria and lactobacilli, while it also promotes the presence of higher bifidobacterial numbers with a specific species composition (Jost et al., 2015). Decreased bifidobacterial abundance may result in reduced lactate/acetate concentrations and increased gut luminal pH, which in turn may cause an aberrant microbiota composition, reduced protection against pathogens, and abnormal immune development with consequent increased allergy risk (Elsen et al., 2019). Human milk contains human milk oligosaccharides (HMOs), which have been shown to promote a bifidobacteria-rich gut community (Aakko et al., 2017). HMOs are not digested by the human host, and will thus reach the intestinal tract, where they may be utilized as a primary carbon and energy source by specific species of the genera *Bifidobacterium* and *Bacteroides* (Brand-Miller et al., 1998; Jost et al., 2015). Based on these characteristics, HMOs are considered natural and highly effective prebiotics for infants. In situations where breast feeding is not possible, infant formula is the most frequently applied feeding alternative. Formula milk preparations are typically based on bovine milk which contains only low levels of bovine milk oligosaccharides of a different composition compared to human milk (Kunz et al., 2000). In order to improve the ability to support colonization and metabolism of bifidobacteria and lactobacilli in the infant gut, such bovine milk based formulations are commonly enriched with enzymatically produced oligosaccharides, in particular galacto-oligosaccharides (GOS) (Jost et al., 2015). GOS is a mixture of oligosaccharides with varying DP (degree of polymerization) and linkages, containing typically a glucose at the reducing end and extended by multiple galactose units. GOS can be enzymatically synthesized by a transgalactosylation reaction employing β-galactosidases of different origin with solely lactose as substrate, where D-galactosyl moieties are transferred onto the D-galactose component of lactose, resulting in addition of a variable number galactosyl moieties and release of glucose as the by product (Torres et al., 2010; Vera et al., 2016). β-galactosidases belong to a class of hydrolytic enzymes (EC 3.2.1.23) which under standard physiological conditions are unable to carry out transgalactosylation activities. For this reason modulation of reaction conditions is necessary to make these enzymes suitable for GOS synthesis. A commonly employed source of industrial GOS synthesis are β-galactosidases of fungal origin (Urrutia et al., 2013; Saqib et al., 2017). However, also bacterial enzymes from *Lactobacillus* species (Splechtna et al., 2006; Wichienchot et al., 2016), *Bacillus circulans* (Vivinal^®^ GOS) (Torres et al., 2010) and *Bifidobacterium* (van Laere et al., 2000; Goulas and Tzortzis, 2007; Han et al., 2014; Viborg et al., 2014), *Streptococcus thermophilus* in combination with *Aspergillus oryzae* (Chen and Gänzle, 2017) have been employed. In the context of infant nutrition, the use of bifidobacterial-derived β-galactosidases is particularly relevant because it may generate GOS preparations that are selectively utilized by *Bifidobacterium* thus possessing bifidogenic properties (Rabiu et al., 2001). GOS is primarily composed of disaccharides and trisaccharides with typically 75–90% ≥ DP2 + DP3, with the most representative trisaccharide being galactosyl-lactose, which is also known to be a (minor) HMO component (Coulier et al., 2009; Newburg et al., 2016). Three different structures of galactosyl-lactose (3′-galactosyllactose, 4′-galactosyllactose, and 6′-galactosyllactose) are present in human colostrum, and have been found to play a role in immunomodulation (Newburg et al., 2016). It has been demonstrated that galactosyl-lactose can physiologically act as a potent anti-inflammatory agent and can positively contribute to immune modulation during infancy. Based on these properties it was suggested that GOS plays a role in protection against enteric inflammation in neonates (Newburg et al., 2016). Other studies have shown that infants nurtured with GOS or GOS/FOS (in a 9:1 w/w ratio)-enriched milk formula harbor an intestinal microbiota with associated metabolic activities that is similar to a microbiota typical of breast-fed infants (Bakker-Zierikzee et al., 2005; Ben et al., 2008; Fanaro et al., 2008; Sierra et al., 2015). GOS preparations, similar to what has been demonstrated for HMOs, have been shown to elicit a protective effect on epithelial cells when tested in a fungal toxin deoxynivalenol (DON)-induced gut barrier disruption model (Akbari et al., 2016; Perdijk et al., 2019). Moreover, GOS was also found to stimulate the production of MUC2, thus indirectly improving the gut barrier function (Figueroa-Lozano et al., 2020). Additionally, GOS has been shown to inhibit the epithelial cell adhesion ability of certain pathogens (Laparra et al., 2013), which is a crucial step prior to invasion of host cells (Ribet and Cossart, 2015). In a recent study administration of varying levels of oligosaccharides prepared by enzymolysis of various polysaccharides from natural sources was shown to significantly reduce adhesion of various pathogenic microorganisms (i.e., *E. coli*, *Vibrio cholerae*, *Campylobacter jejuni*, and *S.* Typhimurium) (Wang et al., 2015). Notably, for *S.* Typhimurium strain SL1344nal it was shown that a particular GOS, produced through lactose-based transgalactosylation mediated by a cell free extract of *Bifidobacterium bifidum* NCIMB 41171, is able to inhibit adhesion of SL1344nal to HT-29 cells (Searle et al., 2009). In addition, various prebiotic oligosaccharides (i.e., galacto-oligosaccharides, inulin, oligofructose) have been demonstrated to inhibit adherence of enteropathogenic *E. coli* to the human cell lines HEp-2 and Caco-2 (Shoaf et al., 2006).

In previous work (Ambrogi et al., 2019) we identified and characterized seventeen β-galactosidases encoded by four different bifidobacterial species, in an attempt to discover potential enzymes for the synthesis of novel GOS with bifidogenic properties. Subsequent investigations demonstrated that seven of the identified enzymes exhibit transgalactosylation activity, thus making these β-galactosidases promising candidates for further characterization (Ambrogi et al., 2019, 2021). In the current work, we describe optimized GOS production by four of the previously characterized bifidobacterial β-galactosidases. We also describe the composition and potential prebiotic functionality of two GOS preparations (designated GOS-A and GOS-E) produced by those β-galactosidases.

## Materials and Methods

### Galacto-Oligosaccharides Production

The enzymes employed in this work are BgaA, BgaC, BgaD, and BgaE, identified and characterized previously (Ambrogi et al., 2019). Basic GOS production was achieved as previously described (Ambrogi et al., 2021) and is briefly outlined as follows: a starting slurry mixture was prepared by mixing 7.89 g of Lactochem^®^ (FrieslandCampina, Amersfoort, Netherlands), 5.1 g of water, 150 μl of 1 M citrate buffer (pH 7), 75 μl of 1 M MgCl_2_. This lactose slurry was allowed to become homogenized for 75 min prior to the addition of a particular β-galactosidase amount diluted in 2 ml of water (the overall suspension then corresponds to a 50% w/w lactose in water mix). The enzymatic reaction to produce GOS was performed in a vessel (Wide neck clear GL50, VWR) with constant stirring at a temperature range between 40 and 55°C. The enzyme reaction was allowed to proceed for no longer than 12 h following enzyme addition. The enzyme dose was varied between 2 and 30 lactase unit (LU) per gram of lactose, with the purpose to investigate the transgalactosylation activity (as judged by the clarification time of the slurry), wherein one LU is defined as the amount of enzyme that is required to release 1 μmol of D-glucose per minute due to lactose hydrolysis (at non-limiting lactose levels) at 10% Lactose in water, 40°C and pH 6.0 (Ambrogi et al., 2021).

The various conditions used to assess GOS production are schematically reported in Table 1. Reaction products were sampled at different time points following the initiation of the GOS synthesis reaction and analyzed by High Performance Anion Exchange Chromatography (HPAEC) for GOS production/content analysis.

**TABLE 1:** Galacto-oligosaccharides assay settings and GOS quantification related to GOS synthesis assay.

Enzyme	Reaction set-up				Reaction out-come		Composition of reaction solutions selected for GOS purification					
			
	Initial lactose%	T (°C)	Starting pH	Enzyme concentration (Lu/g)	Slurry clarification	Best GOS yield on dry matter (%)	Galactose (%)	Glucose (%)	Lactulose (%)	Allo-lactose (%)	Lactose (%)	GOS (%)
BgaC	50	40	6.4	4	No							
		50	6.2	6	No							
		55	6.2	6	No							
	40	55	6.2	4,8,12,16	Partial slurry clarification at 11 h with 16 Lu/g	13.1	11.6	16.7	1.0	0.6	57.0	13.1
BgaD	50	40	6.4	4	no							
		50	6.2	5, 10, 15	Partial slurry clarification at 12 h with 15 Lu/g							
		55	6.2	5, 10, 15	Partial slurry clarification at 5 h with 15 Lu/g							
	40	55	6.2	4,8,12,16	At 6 h with enzyme concentration of 12 and 16 Lu/g/no clarification for reaction with 4 and 8 Lu/g	14.7						
		55	6.2	20, 30	At 5 h in the reaction with 20 Lu and at 4 h for the reaction with 30 Lu/g	22.4	11.28	17.83	1.14	0.84	46.55	22.4
	30	50	6.2	4,8,16,24	After 30 min for all the concentrations tested	15.2						
BgaA	50	50	6.5	3	At 7 h	11.3						
		55	6.2	4, 8, 11	At 2 h for the reaction with 8 Lu and after 90 min for the reaction with 11 Lu/g	19	19.7	29.1	0.5	11.4	20.4	19.0
BgaE	50	50	6.5	3	At 7 h	33.7						
		55	6.2	2,4,6	at 6 h with 2 Lu/g, at 90 min with 4 Lu and at 1 h with 6 Lu/g	40.3	10.3	25.6	0.1	18.1	5.6	40.3

### Galacto-Oligosaccharides Purification

The obtained crude GOS preparation was purified as previously described (Morales et al., 2006) with minor modifications as outlined below. In brief: 50 g of activated charcoal (AC) (Cabot Norit Activated Carbon, Deerlijk, Belgium) was suspended in 500 ml of 0.1 M HCl and allowed to stir for 20 min. The AC suspension was then poured on top of a paper filter (Qualitative filter paper 413, medium filtration rate, particle retention size: 5–13 μm, VWR International), that had been positioned in a Büchner funnel (Büchner funnels, 500 ml, VWR), vacuum dried, after which the AC bed was washed with 500 ml Milli-Q water. Five gram of a given GOS reaction solution was diluted in 100 ml Milli-Q water and added to the AC and allowed to stir for 30 min. Following this, the GOS-loaded AC was washed three times with, respectively, 200 ml Milli-Q water, 300 ml 1% ethanol solution (in order to selectively remove monosaccharides), 300 ml 5% ethanol solution (for the partial removal of lactose) and 35% ethanol solution (aimed at harvesting the oligosaccharides of the sample). The wash solution obtained with the 35% ethanol elution was collected and vacuum centrifuged (Genevac MiVac Quattro sample concentrator, SP Scientific) in order to remove the ethanol. A freeze drier (Savant Modulyo freeze dryer, Millrok Technology) was employed in order to obtain a GOS-enriched powder. The same procedure was used to purify commercial Vivinal^®^ GOS syrup (obtained from FrieslandCampina, Amersfoort, Netherlands).

### Preliminary Galacto-Oligosaccharides Analysis

Separation and visualization of individual saccharide components of GOS preparations were achieved by HPAEC-PAD mounted on a Dionex IC-3000 system. Separations were performed using a CarboPac PA1 (Thermo Scientific) analytical-anion exchange column (dimensions, 250 mm by 4 mm) with a CarboPac PA1 (Thermo Scientific) guard column (dimensions, 50 mm by 4 mm) and a detector (ED40) in the pulsed amperometric detection PAD mode (Dionex, Thermo Scientific). For the chromatographic analysis the following gradient was employed of A: 100 mM NaOH, B: 600 mM NaOAc in 100 mM NaOH, C: Milli-Q water, and D: 50 mM NaOAc as previously described by Van Leeuwen et al. (2016). Quantification of individual saccharidic peaks of a generated chromatogram was performed using CHROMELEON software Ver.7 (Dionex, Thermo Scientific).

### Structural Analysis of Purified Galacto-Oligosaccharides

#### Quantification by Gas Chromatography-Flame Ionization Detector

Carbohydrates were analyzed as their trimethylsilylated oximes (TMSO) according to a previously described method (Brobst and Lott, 1966). Briefly, the oximes were formed by the addition of hydroxylamine chloride in pyridine (2.5% w/v) and heated at 75°C for 30 min, followed by addition of hexamethyldisilazane and trifluoroacetic acid, with subsequent incubation at 50°C for 30 min. Quantification of TMSO derivatives was carried out employing an Agilent Technologies gas chromatograph (Model 7890A) equipped with a flame ionization detector (FID) and the use of a fused silica capillary column DB-5HT (5%-phenyl-methylpolysiloxane; 30 m × 0.25 mm × 0.10 μm) (Agilent, Palo Alto, CA, United States) according to previously reported chromatographic conditions (Julio-Gonzalez et al., 2019).

#### Determination of Molecular Weights by Matrix-Assisted Laser Desorption Ionization Time-of-Flight

Matrix-assisted laser desorption ionization time-of-flight (MALDI-TOF) spectra were recorded employing a Voyager DE-PRO mass spectrometer (Applied Biosystems) equipped with a nitrogen laser emitting at 337 nm with a 3 ns, and 3 Hz frequency. Ions generated by laser desorption were introduced into a time of flight analyzer (1.3 m flight path) with an acceleration voltage of 25 kV, 94% grid voltage, 0.075% ion guide wire voltage, and a delay time of 400 ns in the linear positive ion mode. Mass spectra were obtained over the *m/z* range 100–5000 with 2,5-dihydroxybenzoic acid (>98%, Fluka) being employed as matrix.

#### Characterization by Gas Chromatography-Mass Spectrometry

The TMSO carbohydrate derivatives were analyzed in a gas chromatograph coupled to a quadrupole mass detector (Agilent GC-6890 and MS5973; Agilent, Palo Alto, CA, United States). Column, GC and MS conditions are described previously (Julio-Gonzalez et al., 2019). The identification of TMSO carbohydrate derivatives was carried out by the analysis of corresponding mass spectra and data of previously reported standards (Hernández-Hernández et al., 2011). These identifications were considered as tentative.

#### Isolation by Hydrophilic Liquid Chromatography Coupled to a Refractive Index Detector (HILIC-RID)

Di- and trisaccharides were isolated by semi-preparative HILIC-RID using a ZORBAX NH_2_ column (PrepHT cartridge 250 × 21.2 mm, 7 μm particle size) (Agilent Technologies, Madrid, Spain). Two mL samples of carbohydrate mixture (150 mg/mL) were injected and eluted with acetonitrile (75% v/v) at 21 mL/min. Fractions containing di- and trisaccharides were collected, concentrated in a rotatory evaporator R-200 (Büchi, Flawil, Switzerland) and lyophilized for Nuclear Magnetic Resonance (NMR) spectroscopy analysis (see below).

#### Characterization by Nuclear Magnetic Resonance

Nuclear magnetic resonance spectra were recorded at 298 K, using D_2_O as a solvent, on an Agilent SYSTEM 500 NMR spectrometer (^1^H 500 MHz, ^13^C 125 MHz) equipped with a 5-mm HCN cold probe. Chemical shifts of ^1^H (δ_*H*_) and ^13^C (δ_*C*_) in parts per million were determined relative to internal standards of sodium [2, 2, 3, 3-^2^H_4_]-3-(trimethylsilyl)-propanoate in D_2_O (δ_*H*_ 0.00) and 1,4-dioxane (δ_*C*_ 67.40) in D_2_O, respectively. One-dimensional (1D) NMR experiments (^1^H, and ^13^C) were performed using standard pulse sequences. Two-dimensional (2D) [^1^H, ^1^H] NMR experiments [gradient correlation spectroscopy (gCOSY), and total correlation spectroscopy (TOCSY)] were carried out with the following parameters: delay time of 1 s, spectral width of 3004 Hz in both dimensions, 1024 complex points in t2, 16 transients for each of 200 time increments, and linear prediction to 512. The data were zero-filled to 2048 × 2048 real points. A mixing time of 150 ms was used for TOCSY experiments. 2D [^1^H−^13^C] NMR experiments [gradient heteronuclear single-quantum coherence (gHSQC) and gradient heteronuclear multiple-bond correlation (gHMBC)] used the same ^1^H spectral window, a ^13^C spectral window of 13824 Hz, 1 s of relaxation delay, 1024 data points, and 128 time increments, with a linear prediction to 256. The data were zero-filled to 2048 × 2048 real points. Typical numbers of transients per increment were 16 and 64, respectively.

### Evaluation of Bifidobacterial Growth on Purified Galacto-Oligosaccharides Preparations

Bifidobacterial strains were cultured anaerobically in modified de Man Rogosa and Sharpe (mMRS) medium supplemented with cysteine-HCl (0.05% w/v) and lactose (0.5% v/v) following a previously described method (Ambrogi et al., 2019). An overnight aliquot at 1% (v/v) was inoculated into 5 ml of fresh mMRS medium containing 0.5% final concentration (v/v) of a given purified GOS preparation and 0.05% cysteine-HCl (v/v). The following GOS preparations were used: purified GOS-A, purified GOS-E and a purified commercially available GOS (Vivinal^®^ GOS; FrieslandCampina, Amersfoort, Netherlands). Basal mMRS medium (i.e., without addition of any carbohydrate) was used as a negative control, while mMRS supplemented with lactose (0.5% v/v final concentration) was used as a positive control. Solutions containing glucose, galactose, lactulose, and lactose (hereafter referred to as mono/di-mix) added in the same proportion and amounts as remain present in the corresponding purified GOS preparation were also tested, in order to evaluate bacterial growth supported by the non-GOS components of the substrate (i.e., glucose, lactose, and galactose). For each substrate and control a 5 ml culture was prepared, of which 1 ml was loaded in the 96 well plates, while the remaining 4 ml were incubated anaerobically and used for sample collections for metabolite assessment. Three hundred μl was sampled directly from the tubes at varying time points (6, 8, 12, and 24 h) and stored at –80°C. The 96 well microplates (Microtest Plate 96 well, Sarsted, Germany) were organized as represented in Supplementary Figure S1 and used to evaluate carbohydrate-dependent bacterial growth at 37°C under anaerobic conditions for 24 h. Growth curves were obtained using a 2 Multiskan FC microplate readers (Thermo Scientific) with optical density measurements taken at intervals of 30 minutes and a wavelength of 620 nm. Seven infant-derived bifidobacterial strains (*Bifidobacterium breve* UCC2003, *B. breve* NCFB 11815, *Bifidobacterium bifidum* LMG 11041, *Bifidobacterium longum* subsp. *longum* NCIMB 8809, *B. longum* subsp. *longum* CCUG 3698, *Bifidobacterium longum* subsp. *infantis* ATCC 15697, and *Bifidobacterium pseudocatenulatum* ATCC 27919) and one human adult-derived strain (*Bifidobacterium adolescentis* DSM20083) were tested in triplicate for growth abilities on the various GOS preparations. All have been shown previously to be capable of growth on purified Vivinal^®^ GOS (contains 97% GOS, 0.7% galactose, 1.6% glucose, and 0.7% lactose) (Watson et al., 2013).

The final pH values were recorded from the culture medium of strains after 24 h post-inoculum with a manual pH meter (HI-2210-02, Bench Top pH Meter with pH electrode and °C, HANNA instruments; Leighton Buzzard, United Kingdom).

Significance of differences between growth abilities with Purified Vivinal^®^ GOS and GOS-E was determined using a two-tailed unpaired *t*-test with a confidence level of 95% and presumed significant when the resulting *P*-value was <0.05.

### Metabolite Analysis by High Pressure Liquid Chromatography

In order to quantify the main metabolic end products (i.e., acetate, lactate, and formate) produced by the bifidobacterial strains assessed for growth on purified GOS preparations, samples collected at varying time points during growth were analyzed by high pressure liquid chromatography (HPLC). An aliquot of 300 μl of bacterial culture was filter-sterilized (Corning^®^ Costar^®^ Spin-X^®^ centrifuge tube filters, 0.45 μm Sigma- Corning^®^ Costar^®^) and directly analyzed by means of an Agilent 1200 HPLC system (Agilent Technologies, Santa Clara, CA, United States) with a refractive index detector. Metabolite peaks and concentrations were identified and calculated based on known metabolite retention times and standard solutions at known concentrations. Non-fermented mMRS medium containing carbohydrates served as negative controls. A REXEX 8 μ 8% H organic acid column (300 mm × 7.8 mm, Phenomenex, Torrance, CA, United States) was used and the elution was performed for 25 min with a 0.01 M H_2_SO_4_ solution at a constant flow rate of 0.6 mL/min and temperature at 65°C. The amount of each SCFA was calculated and the lactate/acetate ratio was estimated for all assessed substrates.

### Pathogenic Strains and Growth Conditions

The AIEC *E. coli* strain HM605, which had previously been isolated from a clinical case of gastroenteritis (Clarke et al., 2011), and *S.* Typhimurium strain 4/74, a bovine isolate (Giannella et al., 1973), were used as test microorganisms for adherence inhibition experiments. Before each experiment, cells from a frozen stock of either of the two strains were inoculated (1% v/v) in 10 ml Luria-Bertani (LB) broth grown overnight shaking at 37°C. Since *E. coli* HM605 is resistant to Ampicillin, 10 μg/ml of this antibiotic was added to the overnight culture of this strain for selection purposes. After overnight incubation, the two bacterial cultures were harvested by centrifugation (8000 × *g* for 3 min) and washed three times with Dulbecco’s Phosphate Buffered Saline (PBS) (Sigma Aldrich/Merck). The harvested *E. coli* or *S.* Typhimurium cells were then resuspended to reach a cell concentration of 1 × 10^8^ or 1 × 10^7^ colony-forming units CFU/ml, respectively. A sample of each bacterial suspension was subjected to serial dilution, of which 100 μL volumes were spread on organism-specific selective plates and incubated overnight to confirm viable counts.

### Cell Line Culture

Cell line C2BBe1 (which is a Caco-2 clone; designated as ATCC CRL-2102 from the American Type Culture Collection) was used for *in vitro* adhesion assays according to a previously described method (Corr et al., 2007) with modifications as indicated below. C2BBe1 cells were maintained in a flask containing DMEM supplemented with 10% fetal bovine serum (FBS), 1% penicillin–streptomycin and human apo-Transferrin (0.01 mg/mL Sigma Aldrich/Merck) at 37°C in a 5% CO_2_ atmosphere. Cells were passaged (1: 4 dilution) just before they reached confluency (∼5 days) and the medium was changed every 2 days. Part of the cell culture that was harvested for a new passage was employed for experimental purposes. For this purpose, the C2BBe1 cells were seeded onto a flat-bottomed tissue-culture 24-well plate (Sarstedt) at a concentration of 1 × 10^5^ cells/well (total volume of 1 mL per well). Plates were incubated for 6–7 days at 37°C with 5% CO_2_ until the cells formed a confluent cell layer.

### Pathogenic Bacterial Adhesion Assay

Inhibition assays were modified from two previously described protocols (Shoaf et al., 2006; Searle et al., 2009). Briefly, 500 μl of GOS-containing (final concentration of 0.5, 5, or 25 mg GOS/ml) DMEM supplemented with 10% fetal bovine serum (FBS) and human apo-Transferrin were added to the wells of a 24-well plate. DMEM solutions representing the mono/di-mix control were also added to wells, where they corresponded to the mono/di-saccharides present in the highest GOS concentration (i.e., those present at 25 mg GOS/ml). DMEM and FBS only was used as a negative control. Tissue culture plates were incubated at 37°C in a CO_2_ incubator for 30 min. Bacterial culture was added to each well, and tissue culture plates were then re-incubated at 37°C in a CO_2_ incubator for 30 min. The wells were then gently washed five times with PBS to remove non-adherent bacteria, followed by cell disruption with 0.1% triton (Triton X-100, Sigma Aldrich) solution. To count adherent bacteria, the resulting triton-treated solutions were 10-fold serially diluted 10^0^−10^–3^. The serial dilutions were plated on antibiotic-free LB agar when *S.* Typhimurium was tested and on LB added with ampicillin when *E. coli* was used.

Adhesion experiments were performed in triplicate (technical replicates) and repeated three times (biological replicates). The average of all replicates was calculated and statistically significant differences between the substrate concentrations tested were also analyzed (one-way ANOVA, *p* < 0.05) using Graph Pad Prism version 5 (Graphpad Software, United States).

## Results

### Galacto-Oligosaccharides Production

Four bifidobacterial β-galactosidases, named here as BgaA, BgaC, BgaD, and BgaE, had previously been characterized and evaluated for their transgalactosylation activity in lactose-based slurry reactions (Ambrogi et al., 2021). Various GOS production parameters were evaluated (e.g., selection of optimal temperature, starting lactose level, and initial enzyme concentration) and the resulting GOS preparations were classified into two distinct groups based on their individual HPAEC-PAD-produced oligosaccharide profile. In particular, enzymes BgaC and BgaD were previously shown to synthesize an oligosaccharide mixture with an apparent short-chain profile, whereas BgaA and BgaE generated a long(er)-chain profile (Ambrogi et al., 2021).

The above mentioned four enzymes were further investigated to determine the optimal conditions for GOS synthesis. For this purpose, the enzymatic reaction was started with a high initial-substrate concentration, which represents a saturated lactose solution in which not all available lactose is dissolved and the insoluble lactose can function as a substrate pool, which is able to maintain the relatively constant lactose concentration, prior to the clarification of the whole reaction mixture (Vera et al., 2012). Therefore, clarification time or speed of this slurry during GOS synthesis (i.e., following addition of enzyme) was used as a preliminary indicator of enzymatic reaction progression or kinetics, in which the insoluble lactose is continuously dissolved and acts as a galactose-donor, thus converted into GOS and other by-products, namely mono sugars, i.e., glucose and galactose. Based on this we used the slurry clarification time as one of the selection criteria for the final candidate enzymes and reaction conditions to investigate. Quantitative analysis of the reaction solution, sampled at different time points following slurry clarification, was used to determine GOS concentrations. The transgalactosylation activity of enzymes BgaC and BgaD was initially tested using conditions that were previously found optimal for their hydrolysis activity, corresponding to 40°C and pH 6 (Ambrogi et al., 2019). Ability of hydrolyzing lactose was evaluated based on slurry clarification, indicating ability to hydrolyze enough lactose so as to achieve a clear solution by dissolving all available lactose (Ambrogi et al., 2021). However, under such conditions these two enzymes were unable to achieve slurry clarification and for this reason we tested other conditions. GOS synthesis using BgaC was assessed at higher temperatures (50 and 55°C) and using enzyme levels that varied between 4 and 16 LU per g of lactose, resulting in a final GOS content of 13.1% of the overall carbohydrate content, as determined by quantitative HPAEC analysis (Table 1 and Supplementary Table S1). BgaD transgalactosylation activity was further evaluated with different combinations of initial lactose concentrations (30, 40, or 50%), various reaction temperatures, and employing varying enzyme levels (Table 1). Despite observed slurry clarification at different time points, analysis of some of the reaction samples revealed an increase of galactose and glucose, and a decrease of lactose and GOS (Supplementary Table S2a), indicating that following clarification lactose/GOS hydrolysis dominates over transgalactosylation. In other reaction combinations, galactose and glucose levels were shown to increase with decreasing lactose levels, though GOS levels did not appear to change (Supplementary Table S2a). It is postulated that in this case the enzyme under evaluation exhibits a higher hydrolytic activity toward its substrate and the product (GOS) than its transglycosylation activity, especially when lactose concentration reduces following clarification. Comparable GOS yields were obtained when BgaD was studied at 50°C with 30% initial lactose concentration or 40% initial lactose, at 55°C with an enzyme level between 4 and 16 Lu/g lactose. In this case samples were collected for a longer period of time, between 0.5 and 8 h (Table 1 and Supplementary Tables S2a,b). Partial slurry clarification was observed when the reaction was conducted with 50% initial lactose, indicating that under these conditions insufficient enzyme activity was present to achieve complete clarification. The maximum GOS level obtained with BgaD was 22.4%.

As reported in Table 1 reactions employing 8 and 11 LU BgaA enzyme per g of lactose did lead to slurry clarification at 2 and 1.5 h, respectively. The highest GOS conversion level was obtained with BgaA, equivalent to 19%, and was observed at 3 h in the reaction with 8 LU of this enzyme per g of lactose (Supplementary Table S3), and a similar GOS conversion level was obtained with 11 LU BgaA per g of lactose in 1.5 h (Table 1 and Supplementary Table S3). Samples at time points 1.5 and 6 h were collected and analyzed for both of these reactions, revealing that lactose and GOS levels decreased while glucose and galactose levels increased, suggesting that for these time points the hydrolytic activity of BgaA was dominant over transgalactosylation activity.

In the case of BgaE, slurry clarification was observed for all conditions considered (Table 1). Based on our HPAEC analysis, 4 LU per g of lactose appeared to represent the optimal enzyme level to achieve efficient GOS production, as a relatively minor increase of glucose and galactose was observed over time when compared to the reaction performed with 6 LU BgaE per g of lactose. The highest observed GOS conversion level (40.3%) was obtained at 9 h using 4 Lu BgaE per g lactose, representing the best GOS production results obtained for any of the four enzymes tested (Table 1 and Supplementary Table S4).

### Galacto-Oligosaccharides Purification and Characterization

In order to make samples of Vivinal^®^ GOS as well as GOS produced by BgaA and BgaE (designated here as GOS-A and GOS-E, respectively) suitable for *in vitro* growth experiments, monosaccharides glucose and galactose, and the disaccharide lactose were (at least partially) removed, as described in the Materials and Methods section. GOS mixtures obtained from BgaA at 55°C with an enzyme concentration of 8 LU/gram of lactose and from BgaE at 55°C with an enzyme concentration of 4 LU/gram of lactose were selected for purification. This combination of enzymes and reaction conditions was selected because it resulted in mixtures with at least 19% GOS content and not more than 20% of lactose content.

The resulting two purified GOS preparations and the purified commercially available Vivinal^®^ GOS (PureViv) were then analyzed by HPAEC to determine remaining glucose, galactose and lactose levels and their GOS content. This provided an initial view of their compositional characteristics. The GOS content, which represents the saccharidic fraction of oligosaccharides including allo-lactose, was similar for GOS-E and PureViv at a level of 83.2 and 88.1%, respectively, while for GOS-A the GOS content was 70.4% (Table 2). GOS-A still contained 26.46% lactose, while GOS-E and Purified Vivinal^®^ GOS preparations contained remaining lactose levels of 14.10 and 10.22%, respectively. Traces of monosaccharides were present at similar levels for all three purified products (Table 2). Notably, lactulose, is also present in all three GOS preparations. The presence of lactulose in GOS has been shown previously (Coulier et al., 2009). Based on the fact that the purified crystalline lactose used in this study does not contains detectable amount of lactulose, we assumed that lactulose in the GOS preparations may originate from two main sources. The first is the isomerization of lactose at high temperature and at pH below 7, while the other source is the glucose isomerization into fructose, which consequently be converted to lactulose via beta-galactosidase (Montilla et al., 2005; Song et al., 2013).

**TABLE 2:** Carbohydrate composition of various GOS preparations following AC purification (HPAEC-PAD based quantification express in% on Dry Matter).

Purified GOS	Galactose (%)	Glucose (%)	Allo -lactose (%)	Lactose (%)	Lactulose (%)	GOS (%)	GOS + allo-lactose (%)	Final product amount (g)
GOS-A Purified	1.00	1.52	18.96	26.46	0.62	51.4	70.4	1.00
GOS-E Purified	0.78	1.51	21.87	14.10	0.41	61.3	83.2	5.00
Vivinal GOS Purified	0.36	1.06	2.24	10.22	0.27	85.9	88.1	11.00

### Compositional and Structural Analysis of GOS-A and GOS-E

GOS-A and GOS-E were structurally characterized by a combination of gas chromatography-flame ionization detector (GC-FID), matrix-assisted laser desorption ionization Mass Spectrometry (MALDI-MS), and NMR analysis (see section “Materials and Methods”).

**FIGURE 1:**
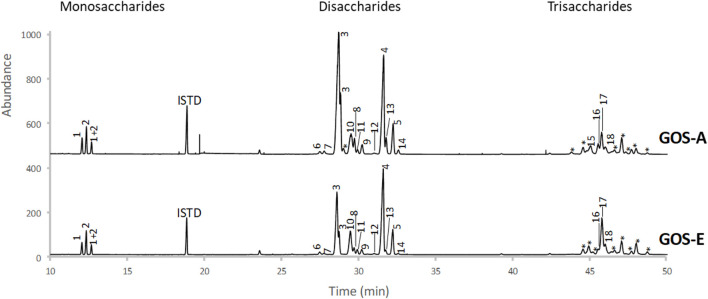
GC-FID profiles of trimethylsilyl oximes of mono-, di-, and trisaccharides from GOS-A and GOS-E. Peak numbers correspond to structures identified in Table 3.

**FIGURE 2:**
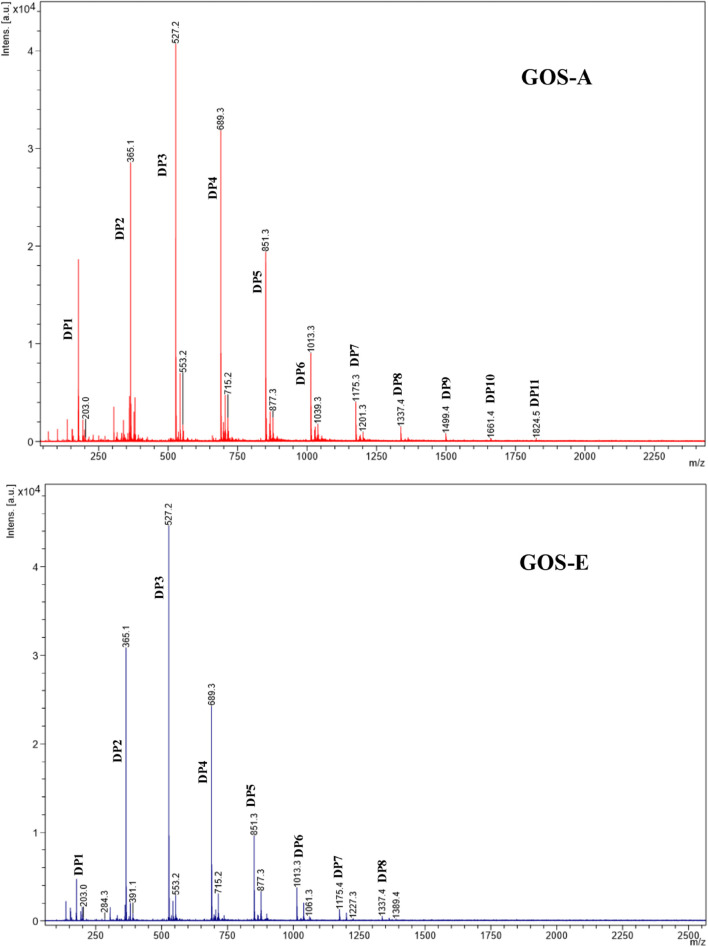
MALDI-ToF mass spectra of GOS-A and GOS-E in the mass range m/z 700–2500. The Degree of Polymerization (DP) are assigned by the molecular weight (M) and their corresponding sodiated adducts [M + Na]^+^.

Based on an initial characterization accomplished by GC-FID (and verified by NMR, see below) the main disaccharide in purified GOS-A was found to be lactose, whereas allo-lactose was the most abundant disaccharide in purified GOS-E. For both GOS preparations, 3′-galactosyl-lactose was shown to be the predominant trisaccharide (Table 3 and Figure 1). In addition, minor levels of 6′-galactosyl-lactose were identified in both GOS samples, whereas 4′-galactosyl-lactose was detected at low levels in GOS-A only. In order to analyze the GOS preparations in terms of oligosaccharides with a degree of polymerization of four or higher, MALDI-MS analysis was performed. The obtained results showed that GOS-E was primarily composed of di-, tri- and tetra-saccharides, consistent with GC-FID analysis (and HPAEC-PAD analysis described above), yet also contained oligosaccharides with a polymerization degree of up to eight. In the case of purified GOS-A oligosaccharides with a degree of polymerization ranging from two of up to 11 were detected (Figure 2).

**TABLE 3:** Di- and tri- saccharides present in GOS-A and GOS-E and corresponding relative%.

	Compound	Peak Number *	GOS-A	GOS-E
Monosaccharides	Galactose	1	1.6	1.7
	Glucose	2	2.3	2.9
Disaccharides	β-D-Gal*p*-(1-4)-D-Glc*p*	3	33.5	17.7
	β-D-Gal*p*- (1-6)-D-Glc*p* E + β-D-Gal*p*- (1-3)-D-Gal*p* Z	4	18.8	21.2
	β-D-Gal*p*- (1-6)-D-Glc*p* Z	5	4.6	5.2
	β-D-Gal*p*- (1-1)-D-Glc*p* E	6	0.5	0.6
	β-D-Gal*p*- (1-1)-D-Glc*p* Z	7	0.7	0.3
	β-D-Gal*p*- (1-2)-D-Glc*p* E	8	5	1.3
	β-D-Gal*p*- (1-2)-D-Glc*p* Z	9	0.6	1.1
	β-D-Gal*p*- (1-3)-D-Glc*p* E	10	0.2	6.6
	β-D-Gal*p*- (1-3)-D-Glc*p* Z	11	2.2	0.9
	β-D-Gal*p*- (1-3)-D-Gal*p* E	12	1.5	0.1
	β-D-Gal*p*- (1-6)-D-Gal*p* E	13	2.1	0.7
	β-D-Gal*p*- (1-6)-D-Gal*p* Z	14	0.6	0.2
	Unknown	*	0.2	0.3
Total disaccharides relative%			**70.3**	**55.9**
Trisaccharides	β-D-Gal*p*- (1-4)-[β-D-gal*p*- (1-4)-]D-Glcp E/Z	15	1.4	0
	β-D-Gal*p*- (1-6)-β-D-gal*p*- (1-4)-Glcp E	16	2	1.3
	β-D-Gal*p*- (1-3)-[β-D-gal*p*-(1-4)-]D-Glcp E	17	4.5	10.6
	β-D-Gal*p*- (1-3)-[β-D-gal*p*-(1-4)-]D-Glcp Z + β-D-(1-6)-Gal*p*-β-D-gal*p*- (1-4)-GlcpZ	18	1.7	2.8
	Unknown	*	11.2	16.7
Total trisaccharides relative%			**20.8**	**31.4**
Total tetrasaccharides relative%	Unknowns	*	**4.7**	**7.7**

*See Figure. Total disaccharide relative % for GOS-A and GOS-E are highlighted in bold.

In order to obtain a more detailed structural characterization of the di- and tri-saccharide fractions, an NMR study was performed by the combined use of 1D and 2D [^1^H, ^1^H] and [^1^H, ^13^C] NMR experiments (gCOSY, TOCSY, multiplicity-edited gHSQC and gHMBC). ^1^H and ^13^C NMR chemical shifts observed are summarized in Table 4.

**TABLE 4:** ^1^H (500 MHz) ^13^C(125 MHz) NMR chemical shifts (δ, ppm) and coupling constants (J in Hz, in parentheses) of compounds 1-5 in GOS-A and GOSE.

		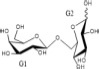		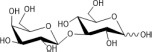		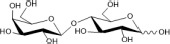		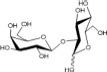		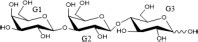	
		Disaccharide 1 b-D-Gal*p*(1→6)D-Glc*p*		Disaccharide 2 b-D-Gal*p*(1→3)D-Glc*p*		Disaccharide 3 b-D-Gal*p*(1→4)D-Glc*p*		Disaccharide 4 b-D-Gal*p*(1→2)D-Glc*p*		Trisaccharide 5 b-D-Gal*p*(1→3) b-D-Gal*p*(1→4)D-Glc*p*	
Fragment	Position	δ^13^C	δ^1^H	δ^13^C	δ^1^H	δ^13^C	δ^1^H	δ^13^C	δ^1^H	δ^13^C	δ^1^H
α –Glc	1	92.77	5.23 (3.7)	92.63	5.24 (3.6)	92.46	5.22 (3.9)	92.54	5.45 (3.6)	92.49	5.23 (3.8)
	2	72.05	3.54	71.55	3.72	71.80	3.58	81.32	3.65	72.06	3.58
	3	73.33	3.72	83.08	3.92	72.06	3.84	72.41	3.85	71.82	3.84
	4	70.05	3.50	68.89	3.53	79.08	3.66	70.05	3.46	78.91	3.66
	5	71.09	4.00	71.78	3.87	70.75	3.95	71.81	3.84	70.75	3.92
	6	69.19	4.17, 3.87	61.19	3.86, 3.80	60.60	3.87, 3.84	61.15	3.84,3.77	60.61	3.88, 3.80
β –Glc	1	96.62	4.66 (8.0)	96.30	4.68 (8.2)	96.40	4.67 (8.0)	95.34	4.73 (8.0)	96.43	4.67 (8.0)
	2	74.66	3.26	74.35	3.43	74.46	3.29	81.96	3.53	74.47	3.29
	3	76.28	3.49	85.32	3.75	75.01	3.66	73.21	3.67	75.44	3.62
	4	70.10	3.49	68.85	3.52	78.95	3.66	70.05	3.44	78.78	3.69
	5	75.33	3.64	76.15	3.50	75.44	3.61	76.54	3.47	75.01	3.65
	6	69.32	4.22, 3.85	61.36	3.92, 3.78	60.73	3.96, 3.80	61.15	3.89,3.70	60.74	3.96, 3.80
Gal-2^α^	1	103.93	4.43 (8.1)	103.97	4.64 (8.1)	103.54	4.45 (7.8)	105.13	4.56 (7.8)	103.17	4.51 (7.8)
	2	71.39	3.55	71.91	3.60	71.62	3.56	71.70	3.59	70.86	3.71
	3	73.30	3.65	73.21	3.69	73.17	3.66	73.27	3.66	82.54	3.84
	4	69.30	3.93	69.22	3.93	69.22	3.93	69.25	3.91	69.09	4.19
	5	75.80	3.70	75.97	3.74	76.00	3.73	75.66	3.69	75.64	3.75
	6	61.65	3.78, 3.75	61.69	3.80, 3.76	61.70	3.80, 3.75	61.60	3.77,3.70	61.67	3.81, 3.76
Gal-2^β^	1	103.93	4.44 (8.2)	104.06	4.66 (8.2)	103.56	4.45 (7.8)	103.98	4.72(7.8)	103.19	4.51(7.8)
	2	71.39	3.55	71.87	3.60	71.61	3.56	71.98	3.57	70.85	3.71
	3	73.30	3.65	73.23	3.69	73.17	3.66	73.21	3.66	82.54	3.84
	4	69.28	3.93	69.22	3.93	69.20	3.93	70.05	3.90	69.07	4.19
	5	75.83	3.70	75.99	3.74	76.00	3.73	76.45	3.68	75.64	3.75
	6	61.64	3.78, 3.75	61.69	3.80, 3.76	61.68	3.80, 3.75	61.60	3.77,3.70	61.65	3.81, 3.76
Gal-1	1									104.99	4.61 (7.7)
	2									71.68	3.60
	3									73.16	3.67
	4									69.23	3.92
	5									75.72	3.70
	6									61.62	3.81, 3.76

#### Galacto-Oligosaccharides-Disaccharide Fraction

The GOS-A and GOS-E disaccharide fraction was separated into three sub-fractions (samples 1, 2, and 3). The 1D ^1^H NMR spectrum of the first sample corresponds to the main identified disaccharide, in both GOS-A and GOS-E (termed as **1** in Table 4), and showed two sets of two doublets in the anomeric region (δ5.23, δ4.43 and δ4.66, δ4.44). In addition, the obtained 1D ^13^C NMR spectrum showed two sets of two resonances in the anomeric region (δ103.93, δ96.62 and δ103.93, δ92.77). These data are compatible with the existence of the two anomeric forms of the reducing terminal unit. The 2D COSY and TOCSY spectra revealed the ^1^H signals of a unit of galactopyranose and a unit of glucopyranose, which allowed us to correlate them with the corresponding carbon signals in the multiplicity-edited gHSQC spectra. To assign the configuration of each anomeric center, the values of the vicinal coupling constants for the anomeric protons were used. These results were consistent with the structure of a disaccharide with the G1 unit being β-D-galactopyranosyl and the reducing terminal G2 unit D-glucose with β and α forms in a 65:35 ratio. The position of the glycosidic linkage was unambiguously accomplished by gHMBC correlations revealing that structure **1** was β-D-Gal*p*-(1→6)-D-Glc*p* (allo-lactose), thereby confirming GC analysis.

Sample 2 corresponds to a disaccharide termed as **2** in Table 4. Using the same procedure as for structure **1**, the 1D ^1^H NMR spectrum showed two sets of two doublets in the anomeric region (δ5.24, δ4.64 and δ4.68, δ4.66). In addition, the 1D ^13^C NMR spectrum showed two sets of two resonances in the anomeric region (δ104.06, δ96.30 and δ103.97, δ92.63). These results in combination with gHMBC correlations allowed the identification of structure **2** as β-D-Gal*p*-(1→3)-D-Glc*p* with β and α forms in a 75:25 ratio.

Finally, sample 3 showed signals for two different disaccharides [termed as **3** (major; relative molar abundance 90%) and **4** (minor; relative molar abundance 10%) in Table 4]. From 2D-experiments and chemical shift values, the major disaccharide was identified as lactose with β and α forms in a 62:38 ratio whilst the minor disaccharide was elucidated as β-D-Gal*p*-(1→2)-D-Glc*p*.

Small quantities of additional, yet minor, disaccharides were detected in the 1D ^1^H NMR and 1D ^13^C NMR spectra of this sub-fraction, resulting in the identification of β-D-Gal*p*-(1→3)-D-Gal*p*, β-D-Gal*p*-(1→1)-D-Glc*p* and β-D-Gal*p*-(1→6)-D-Gal*p* in both GOS-A and GOS-E.

#### Galacto-Oligosaccharides-Trisaccharide Fraction

The 1D ^1^H NMR spectrum of the trisaccharide fraction showed signals of a major compound, (termed as **5** in Table 4), and showed two sets of three doublets in the anomeric region (δ5.23, δ4.51, δ4.61 and δ4.67, δ4.51, δ4.61). In addition, the 1D ^13^C NMR spectrum exhibited two sets of three resonances in the anomeric region (δ104.99, δ103.19, δ96.43 and δ104.99, δ103.17, δ92.49). The 2D COSY and TOCSY spectra revealed the ^1^H signals of two galactopyranose units and a glucopyranose unit. These results were consistent with the structure of a trisaccharide with the G1 and G2 units being β-D-galactopyranosyl, and the reducing terminal unit glucopyranose with β and α forms in a 60:40 ratio. These data combined with gHMBC correlations allowed the identification of structure **5** as β-D-Gal*p*-(1→3)-β-D-Gal*p*-(1→4)-D-Glc*p* (3′-galactosyllactose). The complete ^1^H and ^13^C sets of chemical shifts (Table 4) are identical to those previously identified in Vivinal^®^ GOS (Van Leeuwen et al., 2014).

Table 3 summarizes the quantifiable di- and trisaccharide structures present in GOS-A and GOS-E, as well as their level as a relative percentage of the particular purified GOS preparation. The GOS disaccharide fraction, when ignoring lactose, comprised 36.8% of total GOS in GOS-A and 38.2% in GOS-E, with allolactose representing the main disaccharide in both cases (i.e., 21% and 22% of total GOS, respectively, Table 3). The two tri-saccharides β-D-Gal*p*-(1→6)-β-D-Gal*p*-(1→4)-Glc*p* (6′-galactosyllactose) and β-D-Gal*p*-(1→3)-β-D-gal*p*-(1→4)-D-Glc*p* (3′-galactosyllactose) were found in both GOS-A and GOS-E, while β-D-Gal*p*-(1→4)-β-D-gal*p*-(1→4)-D-Glc*p* (4′-galactosyllactose) was exclusively identified in GOS-A. The relative percentage of the tri-saccharide fraction was 20.8% for GOS-A and 31.4% for GOS-E, whereas the tetrasaccharide fraction represented 4.7 and 7.7% of the overall GOS levels for GOS-A and GOS-E, respectively (Table 3).

Literature-based comparison of GOS-A and GOS-E with (unpurified) Vivinal^®^ GOS showed some distinctive differences between these three GOS preparations: β-D-Gal*p*-(1→1)-D-Glc*p* and β-D-Gal*p*-(1→6)-D-Gal*p* associated with GOS-A and GOS-E were not identified in Vivinal^®^ GOS, while β-D-Gal*p*-(1→4)-D-Gal*p* was not found in GOS-A and GOS-E. All tri-saccharides found in this study were also present in Vivinal^®^ GOS (Table 5; Van Leeuwen et al., 2014).

**TABLE 5:** Comparison of di- and tri-saccharides from Purified GOS-A and Purified GOS-E (NMR analysis described in method) with Vivinal GOS according to Van Leeuwen et al. (2014).

	Compound	GOS-A	GOS-E	Vivinal GOS
Disaccharides	β-D-Galp-(1-6)-D-Glcp	✓	✓	✓
	β-D-Galp-(1-4)-D-Galp			✓
	β-D-Galp-(1-2)-D-Glcp	✓	✓	✓
	β-D-Galp-(1-3)-D-Glcp	✓	✓	✓
	β-D-Galp-(1-3)-D-Galp	✓	✓	✓
	β-D-Galp-(1-1)-D-Glcp	✓	✓	
	β-D-Galp-(1-6)-D-Galp	✓	✓	
Trisaccharides	β-D-Galp-(1-6)-β-D-galp-(1-4)-Glcp	✓	✓	✓
	β-D-Galp-(1-4)-[β-D-galp-(1-6)-] D-Glcp			✓
	β-D-Galp-(1-2)-[β-D-galp-(1-4)-] D-Glcp			✓
	β-D-Galp-(1-2)-[β-D-galp-(1-6)-] D-Glcp			✓
	β-D-Galp-(1-3)-[β-D-galp-(1-6)-] D-Glcp			✓
	β-D-Galp-(1-4)-[β-D-galp-(1-4)-] D-Glcp	✓		✓
	β-D-Galp-(1-3)-[β-D-galp-(1-4)-] D-Glcp	✓	✓	✓
	β-D-Galp-(1-4)-[β-D-galp-(1-2)-] D-Glcp			✓
	β-D-Galp-(1-4)-[β-D-galp-(1-3)-] D-Glcp			✓

*Identified links are identified with a check mark.*

### GOS-E and GOS-A Support Growth of Selected Bifidobacteria

**FIGURE 3:**
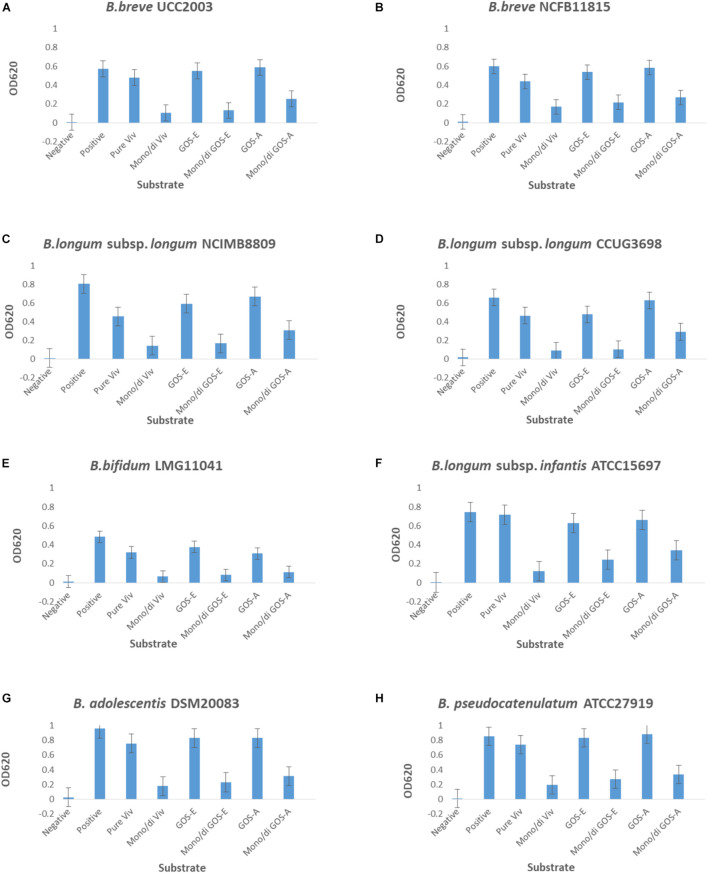
Final OD_620 *nm*_ values after 24 h of growth of **(A)** *S. breve* UCC2003, **(B)** *B. breve* NCFB11815, **(C)** *B. longum* subsp. *longum* NCIMB8809, **(D)** *B*. *longum* subsp. *longum* CCUG3698, **(E)** *B. bifidum* LMG11041, **(F)** *B*. *longum* subsp. *infantis* ATCC15697, **(G)** *B. adolescentis* DSM20083 and **(H)** *B. pseudocatenulatum* ATCC27919. Negative, no sugar; Positive, lactose; Pure Viv, purified Vivinal GOS; Mono/di Viv, Mono/di Mix corresponding to purified Vivinal GOS; GOS-E, purified GOS obtained by enzymatic reaction employing BgaE; Mono/di GOS-E, Mono/di Mix corresponding to GOS-E; GOS-A, purified GOS obtain by enzymatic reaction employing BgaA; Mono/di GOS-A, Mono/di Mix.

The three produced and purified GOS preparations, PureViv (reference), GOS-A (from BgaA) and GOS-E (from BgaE), were assessed for their ability to act as a fermentable carbohydrate source that supports growth of a variety of human-derived bifidobacterial strains. The obtained results showed that all strains were able to grow in a basic mMRS medium containing the positive control carbohydrate lactose, as well as on the three different GOS tested: PureViv as well as GOS-A and GOS-E (Figure 3). In contrast, no appreciable growth (OD_620 *nm*_ ≤ 0.2) was observed in medium containing one of the mono/di mix solutions containing equal amounts of residual glucose, galactose and lactose present in each of the purified GOS preparations. This suggests that the amount of “non-GOS” carbohydrates does not (substantially) obscure the observed GOS-mediated bifidobacterial growth. Among the three mono/di-mix tested, GOS-A-associated mono/di-mix was shown to support a higher level of (residual) growth compared to the mono/di-mix related to GOS-E and Pure Viv. This made it difficult to assess growth differences between GOS-A and the other two GOS preparations. In contrast, due to the very similar levels of residual mono/di-saccharides it was possible to compare the growth results corresponding to purified GOS-E and PureViv. Notably, *B. longum* subsp. *longum* NCIMB 8809 was shown to be able to grow to a higher final OD on GOS-E compared to that obtained for PureViv (*p* < 0.05; Table 6). This observation is in line with previous findings where a given bifidobacterial strain was observed to grow best when a GOS was used that had been produced by a glycosidase derived from that same strain (Rabiu et al., 2001). The final OD values corresponding to the growth of other bifidobacterial strains did not reveal any significant differences between GOS-E and PureViv (Table 6).

**TABLE 6:**
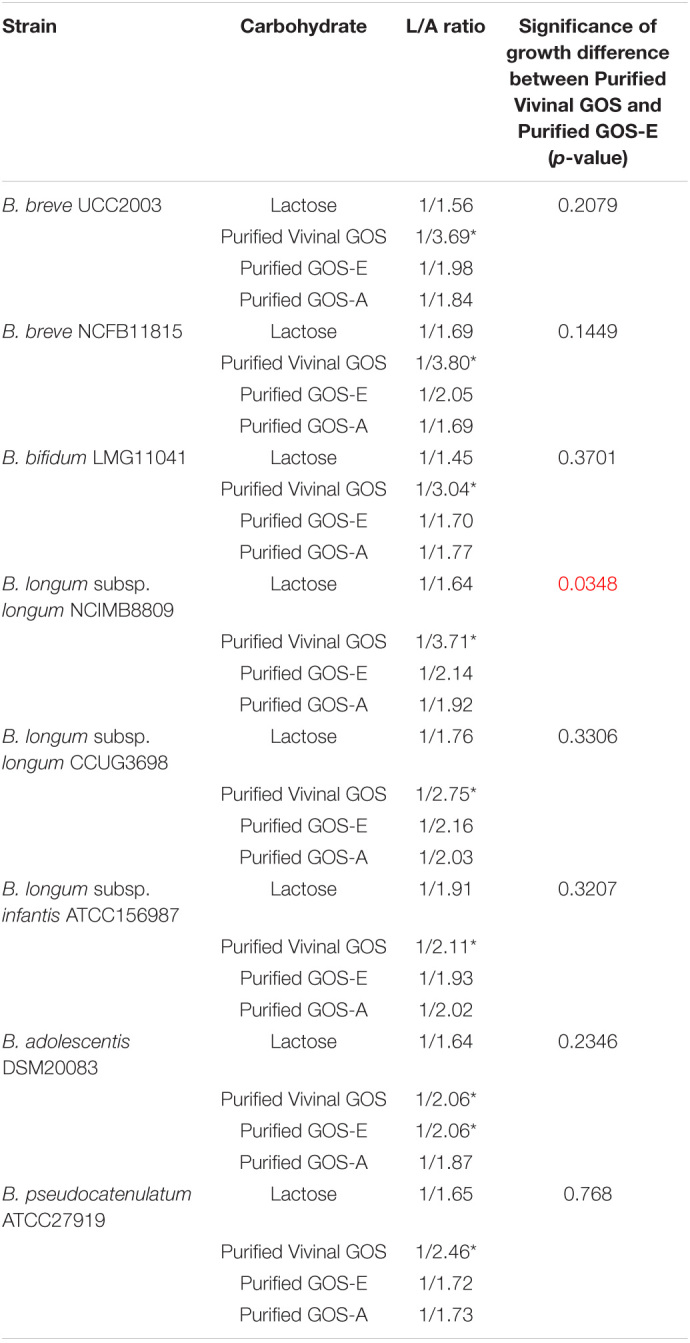
Lactate/Acetate ratio at 24 h of growth experiment and significant bifidobacterial growth.

*Lower L/A ratio. Statistical significance of growth difference based on two-tailed unpaired t-test comparing GOS-E with Vivinal GOS (statistically significant p-values < 0.05). Statistical p-values are indicated in red.

### Short Chain Fatty Acids Production and Acidification

In order to determine the identity and levels of metabolic end products produced by selected bifidobacteria strains during growth on the three purified GOS substrates, we performed HPLC analysis of spent medium. Our results showed that all tested bifidobacteria produced mainly acetate and lactate, with formate being produced at much lower levels. The final concentrations of acetate and lactate (24 h incubation time) were measured and lactate/acetate ratios were calculated (Table 6). When the selected bifidobacterial strains had been cultivated on lactose the determined lactate/acetate ratio was close to the theoretical 1:1.5 (Pokusaeva et al., 2011). Interestingly, the lactate/acetate ratio obtained from bifidobacterial fermentation of PureViv was shown to be the lowest for all strain/substrate combinations tested (Table 6), suggesting that relatively less lactate and relatively more acetate was produced. Notably, despite their distinct composition no major differences were observed in the lactate/acetate ratios resulting from the fermentation of purified GOS-E or GOS-A.

Remarkably, we observed the formation (at low levels) of formate with the highest (final concentration) production observed when PureViv was evaluated as the sole carbon source for all tested strains. In contrast, final lactate concentrations seemed to be higher when bifidobacterial strains were grown on GOS-A or GOS-E when compared to growth on PureViv (Supplementary Figure S2). Overall, we observed that acetate was the metabolite produced at the highest level for all strains tested (Table 6 and Supplementary Figure S2), and across substrates the final concentration of acetate was the highest when bacterial growth was evaluated on lactose. Very similar results between GOS-E and GOS-A were obtained for all strains with the only exception of *B. adolescentis* DSM20083, which produced a lower acetate level when grown on GOS-A (Supplementary Figure S2).

In all cases where a bifidobacterial strain was incubated with lactose (positive control) or any of the three purified GOS preparations (PureViv, GOS-E, or GOS-A) the pH dropped to between 4.5 and 4.8 reflecting acidification in accordance with the observed growth, thus confirming the positive correlation between bifidobacterial growth and acidification (Table 7). Notably, similar and much less acidic pH values were observed for growth on the mono/di-saccharide mixes, while little or no drop in pH was observed, as expected, in the absence of growth in the negative control (no carbohydrate present) (Table 7).

**TABLE 7:** pH values following 24 h growth on Vivinal GOS, GOS-A, GOS-E and related mono/di Mix.

	*B. breve* UCC2003	*B. breve* NCFB11815	*B. bifidum* LMG11041	*B. longum* subsp. *longum* NCIMB8809	*B. longum* subsp. *longum* CCUG30698	*B. longum* subsp. *infantis* ATCC15697	*B. adolescentis* DSM20083	*B. pseudo catenulatum* ATCC 27919
Negative	6.6	6.4	6.4	6.6	6.4	6.6	6.5	6.6
Positive	4.6	4.7	4.6	4.6	4.5	4.5	4.5	4.8
Pure Viv	4.6	4.8	4.8	4.7	4.7	4.6	4.7	4.7
Mono/di Viv	6.3	6.4	6.1	6.2	6.2	6.1	6.2	6.2
GOS-E	4.5	4.8	4.7	4.7	4.6	4.6	4.6	4.7
Mono/di GOS-E	6	5.8	5.6	5.7	5.7	5.9	6	5.9
GOS-A	4.5	4.5	4.6	4.6	4.5	4.5	4.5	4.6
Mono/di GOS-A	5.6	5.5	5.4	5.5	5.4	5.5	5.6	5.6

*Negative: no sugar; Positive: lactose; Pure Viv, purified Vivinal^®^ GOS; Mono/di Viv, Mono/di Mix corresponding to Pure Viv; GOS-E, purified GOS obtained by enzymatic reaction employing BbgE; Mono/di GOS-E, Mono/di Mix corresponding to purified GOS-E; GOS-A, GOS-A obtained by enzymatic reaction employing BbgA; Mono/di GOS-A, Mono/di Mix corresponding to GOS-A.*

### Inhibition of Pathogen Adhesion by GOS-E and Vivinal^®^ Galacto-Oligosaccharides

**FIGURE 4:**
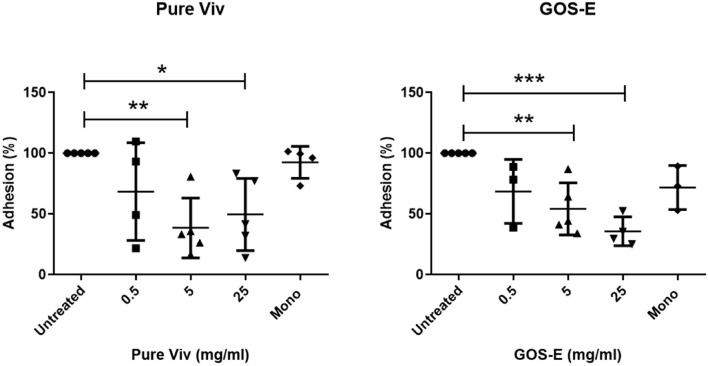
Anti-adhesion experiments. Inhibition of *E*. *coli* HM605 adherence to C2BBe1 tissue culture cells by Pure Viv **(left panel)** or GOS-E **(right panel)**. The data were collected by counting the number of viable *E. coli* HM605 cells following C2BBe1 cell culture disruption. Significant differences were found for 5 and 25 mg/ml tests for both GOS substrates (see Table 9). Lines represent the standard deviations of the means; *n* = 5. The asterisks indicate statistically significant differences: *P ≤ 0.05; **P ≤ 0.01; ***P ≤ 0.001.

In order to investigate the potential ability of GOS-E to inhibit adhesion of pathogenic bacteria to the human intestinal cell line C2BBe1, the latter cell line was exposed to *E. coli* HM605 or *S.* Typhimurium 4/74 in presence of either 0.5, 5, and 25 mg/ml of purified Vivinal^®^ GOS (Pure Viv) or GOS-E. The results of the adhesion assay revealed a significant decrease of *E. coli* HM605 adhesion in the presence of either PureViv or GOS-E when compared to the untreated group without GOS (Table 8 and Figure 4). Notably, addition of 5 or 25 mg/ml of PureViv significantly reduced *E. coli* adhesion by 61.49 and 50.41%, respectively (Table 9), while 0.5 mg/ml of PureViv did not significantly reduce bacterial adhesion (Table 9). When the Mono/di-saccharide mix corresponding to PureViv (Mono/di Viv) was used, bacterial adhesion did not significantly decrease, indicating that these mono/di-saccharides at the concentration tested (and representing 12% of PureViv; Table 2) do not significantly interfere with *E. coli* adhesion (Table 9).

**TABLE 8:** One-way analysis of variance (ANOVA) Dunnett’s multiple comparison test (*P*-value).

Test	*P*-value*
Pure Viv	0.0050
GOS-E	0.0007

*The values represent the statistical analysis summary of the comparison between the obtained adhesion inhibition values, for different concentrations of a given tested GOS preparation, and the untreated test (see Table 9).

**TABLE 9:** Anti-adhesion activity of PureViv and GOS-E at different concentrations.

	Test*			
	0.5	5	25	Mono-mix
Mean difference PureViv	31.59 ± 1.873	61.49 ± 3.867	50.41 ± 3.170	7.424 ± 0.4402
*P*-value PureViv	0.2755	0.0045	0.021	0.9874
Mean difference GOS-E	31.43 ± 2.535	45.81 ± 4.267	64.3 ± 5.646	28.22 ± 2.276
*P*-value GOS-E	0.0884	0.0027	0.0002	0.1433
Significant difference	No	Yes	Yes	No

*Inhibition of E. coli HM605 adherence to C2BBe1 tissue culture cells. Comparison between adherence levels (expressed in%) obtained when the two GOS preparations (at 0.5, 5, 25 mg/ml GOS, as indicated), the Mono-mix or negative control (no sugars added, representing 100% adherence) were tested. Differences were considered statistically significant when P < 0.05. Results were generated by One-way analysis of variance as reported in Table 8.

When GOS-E was tested, a dose-dependent trend was observed. Bacterial adhesion decreased significantly by 45.81 and 64.3% when 5 or 25 mg/ml GOS-E was employed, respectively (Table 2). When GOS-E was tested at a concentration of 0.5 mg/ml, reduction of adhesion (31.4%) was observed.

The results related to *E. coli* adhesion inhibition obtained in this study showed that GOS-E and PureViv both inhibit adherence of AIEC to the surface of tissue culture cells. In contrast to the observations made for *E. coli* HM605, experiments with *S.* Typhimurium 4/74 did not generate consistent results (Supplementary Table S5). The integrity of C2BBe1 cells following exposure to *S.* Typhimurium 4/74 (when compared to C2BBe1 cell integrity following exposure to *E. coli*), looked compromised and formation of microscopic bubbles was observed (Supplementary Figure S3. Previous work has shown that *Salmonella* adhesion using intestinal cell models can very much depend on the serovar and the cell model used (Gagnon et al., 2013). Our observations suggest that the C2BBe1 model is not suitable for this assay and confirm that *S.* Typhimurium 4/74 is not highly adherent, yet is highly invasive (Cossart and Pizarro-cerda, 2006).

## Discussion

In the current study, four β-galactosidases, BgaA, BgaC, BgaD, and BgaE, originating from infant-derived bifidobacterial species *B. breve*, *B. bifidum*, and *B. longum* subsp. *longum* were studied and optimized for GOS synthesis. In our study, we used the clarification time as an indirect measure of the rate at which the enzyme converts lactose into GOS. While starting from a slurry might not be the preferred approach to fully optimize the GOS reaction and yields for each specific enzyme, the clarification time and the resulting GOS composition allowed us to use this simplified approach to evaluate the GOS synthesis rate and the GOS profile and GOS composition of the GOS mixtures obtained from different bifidobacterial enzymes and study their characteristics. The two most promising novel GOS preparations, GOS-E and GOS-A, were further characterized and first indications for bifidogenic potential and anti-pathogenic properties were obtained. The main factors that are known to influence β-galactosidase-mediated GOS production are pH, temperature, water activity and (when fully dissolved) initial lactose concentration. It has previously been demonstrated that a high initial lactose concentration favors enzyme stability (Warmerdam et al., 2013), decreases water activity, increases availability of glycosyl acceptors and consequently facilitates more efficient GOS synthesis (Mahoney, 1998; Gosling et al., 2011). Increased temperature was also shown to support (for some β-galactosidases) higher transgalactosylation activity and consequent enhanced GOS production levels (Gosling et al., 2011).

We showed that an initial lactose concentration of 50% (although not fully dissolved at the start, but clarified during the enzyme reaction) and high temperature represents a suitable combination to achieve a GOS yield of 40.3 and 19% when employing the BgaA and BgaE β-galactosidases, respectively. These results are in line with reported GOS yields of 26 and 47% when bifidobacteria-derived crude β-galactosidases preparations were used for galacto-oligosaccharides synthesis (Rabiu et al., 2001). Our findings demonstrate that despite testing various conditions, BgaC and BgaD are unable to shift their hydrolytic activity toward a more efficient transgalactosidic activity, indicating that transgalactosylation activity is enzyme specific as also shown previously (Oh et al., 2017).

Structural characterization of GOS-A and GOS-E revealed that the predominant (non-lactose) disaccharide was allo-lactose in both cases. Formation of this disaccharide probably occurs because glucose is the preferred acceptor for the galactosyl transfer compared to galactose or lactose, a finding which is in accordance with a previous publication (Arreola et al., 2014). The predominant trisaccharide in GOS-A and GOS-E was shown to be 3′-galactosyllactose. In addition, minor levels of 6′-galactosyllactose were found in both GOS-A and GOS-E, while GOS-A was in addition shown to contain 4′-galactosyllactose. Interestingly, all these three oligosaccharides have also been found in human milk (Newburg et al., 2016).

Comparison between GOS-A, GOS-E, and the unpurified commercial Vivinal^®^ GOS linkages (from an earlier study, Van Leeuwen et al., 2014) showed differences in the di- and trisaccharide fraction (Table 5). Most of the disaccharides found in GOS-A and GOS-E are also present in Vivinal^®^ GOS with the apparent exception of two minor disaccharides [β-D-Galp-(1-1)-D-Glcp and β-D-Galp-(1-6)-D-Galp]. All trisaccharides detected in GOS-A and GOS-E have also been reported to be present in Vivinal^®^ GOS. Based on this previous work it seems that Vivinal^®^ GOS contains a higher variety of trisaccharide structures than GOS-A and GOS-E, though this outcome might also be due to the fact that our structural analyses did not represent an exhaustive attempt to identify all carbohydrates present.

The identification of β-galactosyl residues connected by 1→4, 1→6, 1→3, and 1↔1 β-glycosidic linkages represent a positive and important role for the understanding of the prebiotic potential of the GOS obtained in this study. Previous studies have shown that bifidobacteria are able to grow on di- and tri-saccharides containing these β-glycosidic linkages (van Laere et al., 2000; Cardelle-Cobas et al., 2011).

Bifidobacteria are known to metabolize different types of oligosaccharides, among which galacto-oligosaccharides (Palframan et al., 2003; Watson et al., 2013; James et al., 2016; Thongaram et al., 2017; Arboleya et al., 2018). In this study GOS-A, GOS-E and Pure Viv were found to support growth of all tested bifidobacterial strains. Significantly more growth was observed for *B. longum* subsp. *longum* NCIM8809 when GOS-E was the sole carbon source compared to PureViv. Notably, GOS-E is produced by BgaE which is one of the β-galactosidases from *B. longum* subsp. *longum* NCIM8809.

The major metabolic end products from all carbohydrate fermentations with all *Bifidobacterium* strains were, as expected, acetic acid, lactic acid, and formic acid, with acetate reaching the highest concentrations. Carbohydrate metabolism through the bifid shunt results in a theoretical lactate: acetate molar ratio of 1.5/1 from the fermentation of hexose sugars. In this study we found ratio’s ranging from 1/1.56 to 1/3.04 depending on the different strain-carbohydrate combinations, which is in line with previous demonstrations that the theoretical lactate/acetate molar ratio for bifidobacterial strains is not always observed (Palframan et al., 2003; De Vuyst et al., 2013; McLaughlin et al., 2015). This is due to the fact that pyruvate can not only be reduced to lactic acid, but can also be converted into formate and acetate via the bifid shunt in order to generate additional ATP (Van Der Meulen et al., 2006). Our results indicated that acetate was the metabolite produced at the highest level for all strains tested, with acetate concentration being at the highest when bacterial growth was evaluated on lactose. This confirms what had previously been observed by other authors and reflecting the highest final optical densities achieved when strains where grown on this substrate (Van Der Meulen et al., 2006; De Vuyst et al., 2013). We also observed formate production, which is associated with a higher energy requirement when bifidobacteria ferment complex carbohydrates as it provides additional ATP to support bacterial growth and acetate production compared to growth on simple carbohydrates (Rivière et al., 2016). The two purified GOS preparations obtained in this study promoted growth and metabolic activity of different bifidobacterial species in a similar manner as compared to a commercially available purified GOS. It has previously been reported that high production of acetate in association with low production of lactate (and formate production) in bifidobacteria is associated with a low metabolic rate (De Vuyst et al., 2013). Based on this we speculate that consumption of Pure Viv was somewhat slower compared to the consumption of the other purified GOS tested in this experiment.

Furthermore, we demonstrated anti-pathogenic potential of the novel GOS-E preparation and the purified commercial prebiotic Vivinal^®^ GOS by showing reduced adhesion of *E. coli* HM605 on the C2BBe1 intestinal epithelial cell line *in vitro*, as described previously (Shoaf et al., 2006). The adhesive activity of pathogens to epithelial cells very much depends on their interaction with specific structures on the epithelial cell surface and on the expression of corresponding adherence factors (i.e., adhesins). The similarity between certain oligosaccharide structures and host cell surface receptors, to which bacteria attach prior to invasion, has been taken as the reason for the anti-adherence effect of (galacto-)oligosaccharides (Quintero-villegas et al., 2013). In line with this notion is the finding that the interaction between oligosaccharides and bacterial adhesins is pathogen-specific and strain-specific (Quintero et al., 2011). Further investigations will be needed in order to obtain a better understanding of the mechanism of the observed inhibition, also combined with similar tests employing different pathogenic microorganisms in order to target a more broad range of susceptible bacteria.

## Conclusion

The work described in this paper represent an important contribution toward the use of individually purified bifidobacterial β-galactosidase enzymes for GOS production using lactose as a carbohydrate feeding stock. The research of candidate prebiotics, which have the potential to specifically enhance the abundance and/or metabolic activity of certain bacterial species, is expected to continue in the coming years. In this context, our findings pertaining to a novel GOS that has the ability to support bifidobacterial growth represents a promising start in its potential as a prebiotic.

## Data Availability Statement

The original contributions presented in the study are included in the article/Supplementary Material, further inquiries can be directed to the corresponding author/s.

## Author Contributions

MS and DS conceived the study. VA, JM, LC, and BK designed the experiments. VA, JB, ED, EO’K, OH-H, and BS carried out the experiments. VA, DW, ED, OH-H, and BS analyzed the data. VA, FB, JM, MJ, OH-H, FM, DS, MS, LC, BK, and BS wrote the manuscript. All the authors discussed the results and commented on the manuscript.

## Conflict of Interest

MS, BK, LC, BS, and LC are employees of FrieslandCampina. The remaining authors declare that the research was conducted in the absence of any commercial or financial relationships that could be construed as a potential conflict of interest.

## Publisher’s Note

All claims expressed in this article are solely those of the authors and do not necessarily represent those of their affiliated organizations, or those of the publisher, the editors and the reviewers. Any product that may be evaluated in this article, or claim that may be made by its manufacturer, is not guaranteed or endorsed by the publisher.
