# Supplementary material for: Bifidobacterial β-Galactosidase-Mediated Production of Galacto-Oligosaccharides: Structural and Preliminary Functional Assessments
Source: Front Microbiol. 2021 Oct 28;12:750635. doi: 10.3389/fmicb.2021.750635 (PMC8581567; doi:10.3389/fmicb.2021.750635)
Supplement: Supplementary file 1 [file Data_Sheet_1.PDF]

Supplementary material

Supplementary Table S1. GOS sample quantification from GOS assay related to BgaC experiment

Enzyme	galactose (%)	glucose (%)	allo-lactose (%)	lactose (%)	lactulose (%)	GOS (%)
**BgaC 12Lu**	9.83	14.48	0.44	65.07	1.51	8.7
**BgaC 16Lu**	11.61	16.68	0.57	57.02	1.00	13.1

Supplementary Table S2a GOS sample quantification from GOS assay related to BgaD experiment 40% lactose, 55°C, 12, 16, 20, 30 Lu/g

Enzyme	galactose (%)	glucose (%)	allo-lactose (%)	lactose (%)	lactulose (%)	GOS (%)
**BgaD 12Lu 08h**	12.68	20.05	1.01	51.52	1.24	13.5
**BgaD 12Lu 10h**	14.67	22.62	1.28	47.42	1.05	13.0
**BgaD 12Lu 12h**	15.46	23.41	1.54	43.76	1.16	14.7
**BgaD 16Lu 08h**	14.76	22.68	1.30	46.41	1.15	13.7
**BgaD 16Lu 10h**	16.85	25.23	1.72	41.65	1.04	13.5
**BgaD 16Lu 12h**	17.65	25.96	1.71	38.07	1.07	15.5
**BgaD 20LU 5H**	10.72	17.29	0.64	55.40	1.35	14.6
**BgaD 20LU 6H**	11.61	18.40	0.74	50.83	1.18	17.2
**BgaD 20LU 7H**	12.45	19.54	0.84	48.96	1.18	17.0
**BgaD 20LU 8H**	13.42	20.87	0.93	46.50	1.10	17.2
**BgaD 30LU 4H**	11.28	17.83	0.84	46.55	1.14	22.4
**BgaD 30LU 5H**	14.05	21.38	1.02	41.89	1.03	20.6
**BgaD 30LU 6H**	15.05	22.56	1.18	38.73	0.97	21.5
**BgaD 30LU 7H**	16.51	24.35	1.35	37.45	0.98	19.4
**BgaD 30LU 8H**	17.61	25.50	1.50	34.67	0.86	19.8

Supplementary Table S2b GOS sample quantification from the GOS producing assay corresponding to BgaD used at 4, 8, 16, 24 Lu/g lactose, with 30% lactose, and performed at 55°C.

	galactose (%)	glucose (%)	allo-lactose (%)	lactose (%)	lactulose (%)	GOS (%)
**BgaD 4LU-0.5h**	0.47	1.23	n.a.	92.71	1.65	3.9
**BgaD 4LU-1h**	0.78	1.96	n.a.	88.46	1.78	7.0
**BgaD 4LU-2h**	1.62	3.58	n.a.	84.35	1.46	9.0
**BgaD 4LU-3h**	2.53	5.19	n.a.	82.61	1.73	7.9
**BgaD 4LU-4h**	3.01	6.00	n.a.	78.97	1.42	10.6
**BgaD 4LU-5h**	3.54	6.89	n.a.	78.56	1.61	9.4
**BgaD 4LU-6h**	4.28	7.95	n.a.	76.01	1.36	10.4
**BgaD 4LU-8h**	4.81	8.75	0.07	73.89	1.27	11.2
**BgaD 8LU-0.5h**	1.20	2.77	n.a.	87.36	1.46	7.2
**BgaD 8LU-1h**	2.10	4.41	n.a.	81.55	1.32	10.6
**BgaD 8LU-2h**	4.25	7.89	n.a.	75.17	1.22	11.5
**BgaD 8LU-3h**	6.21	10.73	0.15	68.59	1.29	13.0
**BgaD 8LU-4h**	7.43	12.24	0.22	65.48	1.04	13.6
**BgaD 8LU-5h**	8.59	13.84	0.27	62.47	1.37	13.5
**BgaD 8LU-6h**	9.99	15.53	0.55	59.73	1.31	12.9
**BbglIV 8LU-8h**	11.33	17.21	0.44	57.65	0.87	12.5
**BgaD 16LU-0.5h**	2.50	5.14	n.a.	81.64	0.95	9.8
**BgaD 16LU-1h**	4.31	8.05	0.08	75.33	0.98	11.2
**BgaD 16LU-2h**	11.06	16.90	0.46	55.42	1.14	15.0
**BgaD 16LU-3h**	8.08	13.29	0.27	64.45	1.09	12.8
**BgaD 16LU-4h**	13.05	19.36	0.60	52.67	0.79	13.5
**BgaD 16LU-5h**	14.73	21.27	0.73	48.55	0.93	13.8
**BgaD 16LU-6h**	16.78	23.46	1.03	45.05	0.94	12.7
**BgaD 16LU-8h**	18.37	25.25	1.20	41.26	0.82	13.1
**BgaD 24LU-0.5h**	3.81	7.37	n.a.	78.00	1.17	9.6
**BgaD 24LU-1h**	6.31	10.99	0.15	69.18	0.99	12.4
**BgaD 24LU-2h**	11.17	17.07	0.48	55.67	0.80	14.8
**BgaD 24LU-3h**	15.10	21.55	0.83	47.19	0.88	14.5
**BgaD 24LU-4h**	17.10	23.76	0.97	42.14	0.85	15.2
**BgaD 24LU-5h**	19.46	26.44	1.17	39.29	0.70	12.9
**BgaD 24LU-6h**	21.56	28.50	1.36	35.21	0.75	12.6
**BgaD 24LU-8h**	23.77	30.90	1.55	32.06	0.64	11.1

Supplementary Table S3. GOS sample quantification from the GOS production assay corresponding to BgaA used at 4, 8 and 11 Lu/g lactose, with 50 % lactose as starting substrate level, and performed at 50°C.

Enzyme	galactose (%)	glucose (%)	allo-lactose (%)	lactose (%)	lactulose (%)	GOS (%)
**BgaA 4Lu 6h**	2.64	5.81	2.43	75	1.22	12.9
**BgaA 8Lu 1.5h**	13.29	21.49	10.12	40.11	0.75	14.2
**BgaA 8Lu 3h**	19.66	29.08	11.43	20.39	0.48	19
**BgaA 8Lu 5h**	26.78	36.5	10.41	9.22	0.29	16.8
**BgaA 8Lu 6h**	29.04	38.69	9.48	6.57	0.18	16
**BgaA 11Lu 1.5h**	17.46	26.47	11.14	25.37	0.56	19
**BgaA 11Lu 3h**	26.9	36.87	10.36	9.42	n.a.	16.5
**BgaA 11Lu 5h**	34.43	43.74	6.91	2.47	n.a.	12.5
**BgaA 11Lu 6h**	35.83	44.72	5.84	1.55	n.a.	12.1

Supplementary Table S4. GOS sample quantification from the GOS production assay corresponding to BgaE used at 2, 4 and 6 Li.g lactose, with 50% lactose, and performed at 50 °C.

Enzyme	galactose (%)	glucose (%)	allo-lactose (%)	lactose (%)	lactulose (%)	GOS (%)
**BgaE 2Lu 05h**	3.6	12.11	9.93	50.24	1.04	23.1
**BgaE 2Lu 10h**	4.61	15.13	13.22	36.58	0.6	29.9
**BgaE 2Lu 12h**	4.99	16.19	14.32	33.16	0.58	30.8
**BgaE 4LU 3h**	6.07	18.98	17.01	24.94	0.44	32.6
**BgaE 4LU 5h**	7.88	22.65	19.17	13.5	0.33	36.5
**BgaE 4LU 7h**	9.76	25.63	19.64	7.93	0.22	36.8
**BgaE 4LU 9h**	10.31	25.58	18.09	5.63	0.13	40.3
**BgaE 6LU 1.5h**	5.21	16.87	15.21	32.75	0.67	29.3
**BgaE 6LU 3h**	7.66	22.23	19.16	13.97	0.33	36.6
**BgaE 6LU 5h**	10.06	25.94	19.17	6.62	0.19	38
**BgaE 6LU 7h**	12.6	29.28	18.16	4.14	0.08	35.7

Supplementary Table S5 Adhesion experiment conducted with *Salmonella typhimurium* 4/74 and PureViv.

	**Replicates (%)**		
**Substrate**	**I**	**II**	**III**
**Control**	100.0	100.0	100.0
**0.5 %**	211.4	468.7	119.1
**5 %**	125.6	522.3	70.7
**25 %**	79.7	406.7	646.5
**Mono Mix**	0.0	72.9	80.3

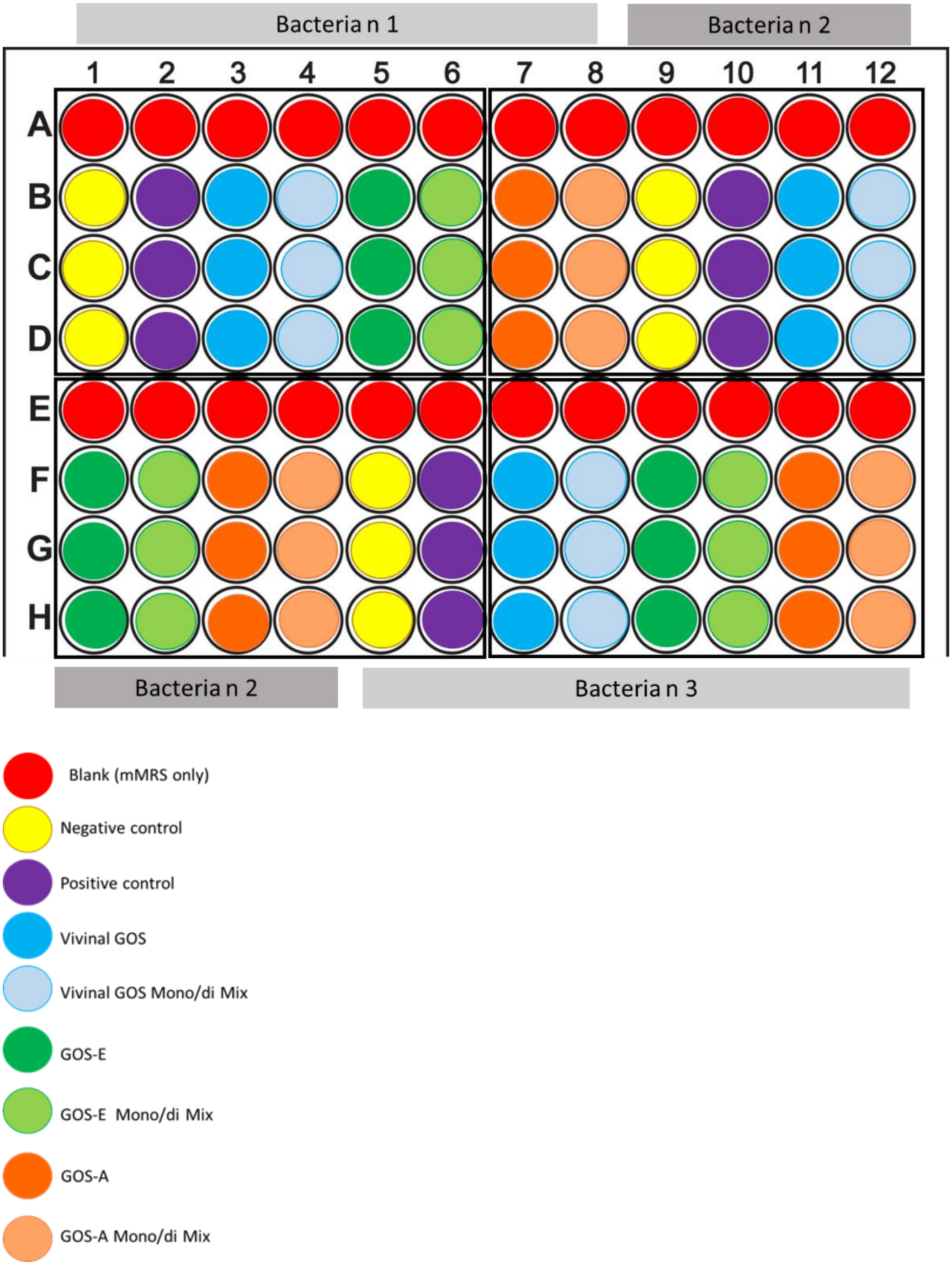

Supplementary Figure S1. 96 wells plate set up for bacterial growth assay.

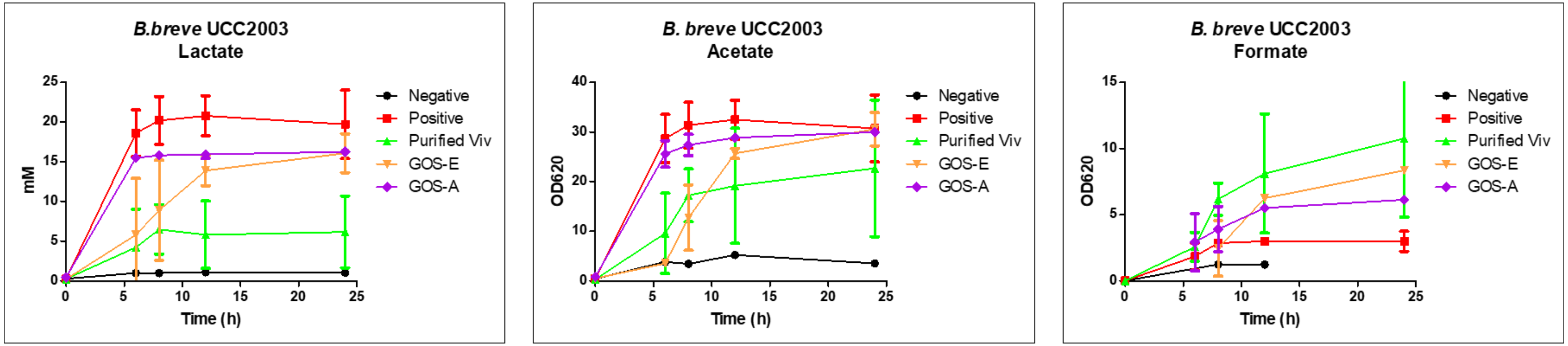

Supplementary Figure S2.a SCFA production resulting from *B. breve* UCC2003 growth on novel purified GOS preparations. Negative: no sugar, Positive: lactose, Purified Viv: Pure Viv, GOS-E: purified GOS obtained by enzymatic reaction employing BgaE, GOS-A: purified GOS obtain by enzymatic reaction employing BgaA.

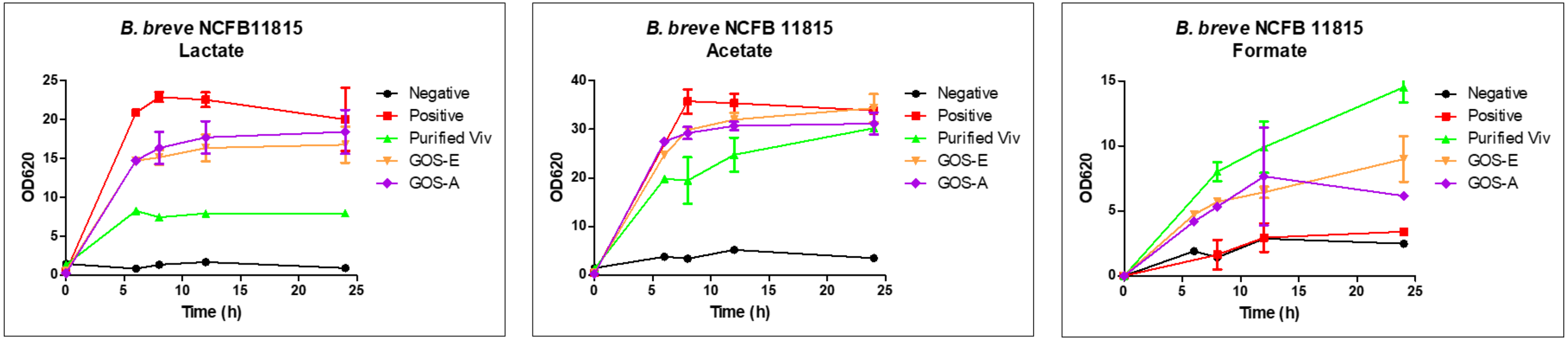

Supplementary Figure S2.b SCFA production resulting from *B. breve* NCFB11815 growth on novel purified GOS preparations. Negative: no sugar, Positive: lactose, Purified Viv: Pure Viv, GOS-E: purified GOS obtained by enzymatic reaction employing BgaE, GOS-A: purified GOS obtain by enzymatic reaction employing BgaA.

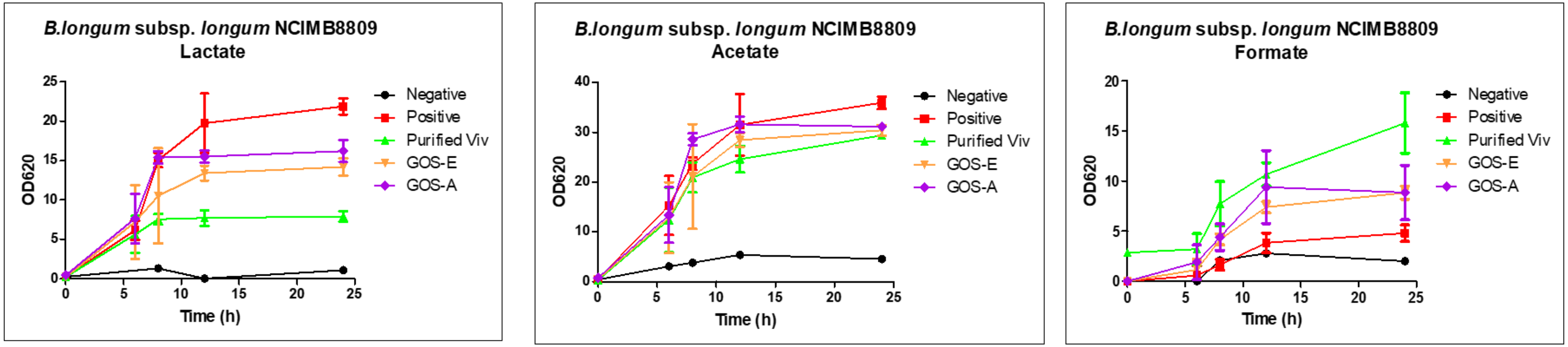

Supplementary Figure S2.c SCFA production resulting from *B. longum* subsp. *longum* NCIMB8809 growth on novel purified GOS preparations. Negative: no sugar, Positive: lactose, Purified Viv: Pure Viv, GOS-E: purified GOS obtained by enzymatic reaction employing BgaE, GOS-A: purified GOS obtain by enzymatic reaction employing BgaA.

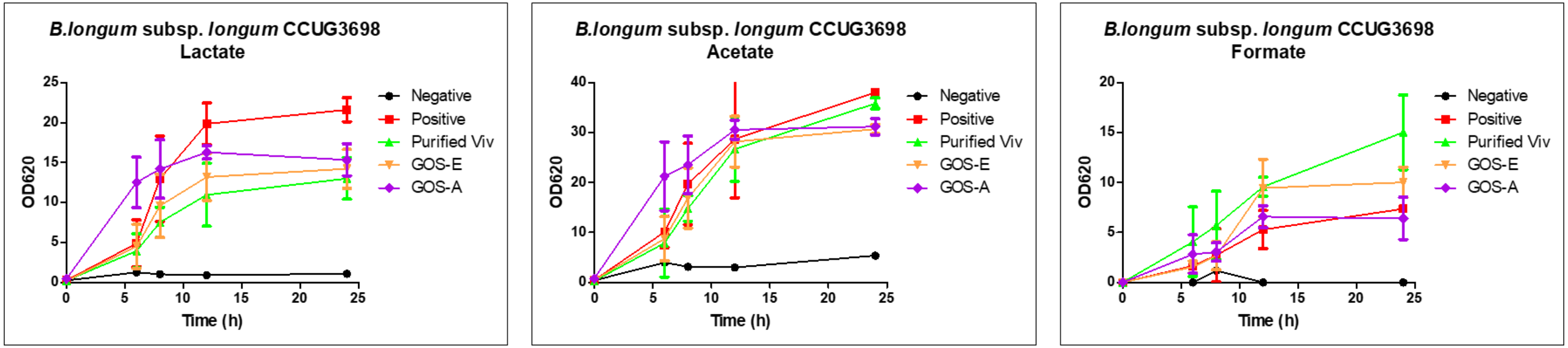

Supplementary Figure S2.d SCFA production resulting from *B. longum* subsp. *longum* CCUG3698 growth on novel purified GOS preparations. Negative: no sugar, Positive: lactose, Purified Viv: Pure Viv, GOS-E: purified GOS obtained by enzymatic reaction employing BgaE, GOS-A: purified GOS obtain by enzymatic reaction employing BgaA.

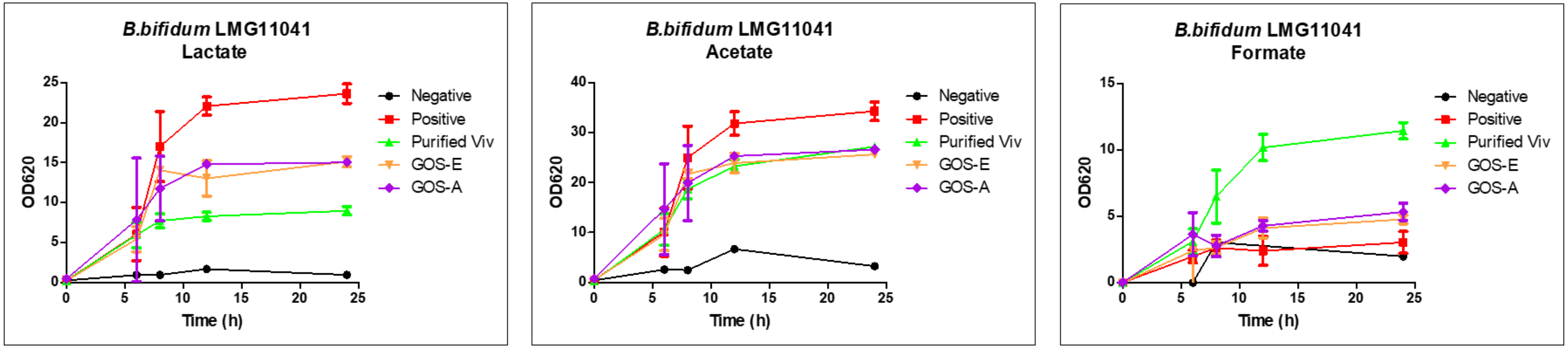

Supplementary Figure S2.e SCFA production resulting from *B.bifidum* LMG11041 growth on novel purified GOS preparations. Negative: no sugar, Positive: lactose, Purified Viv: Pure Viv, GOS-E: purified GOS obtained by enzymatic reaction employing BgaE, GOS-A: purified GOS obtain by enzymatic reaction employing BgaA.

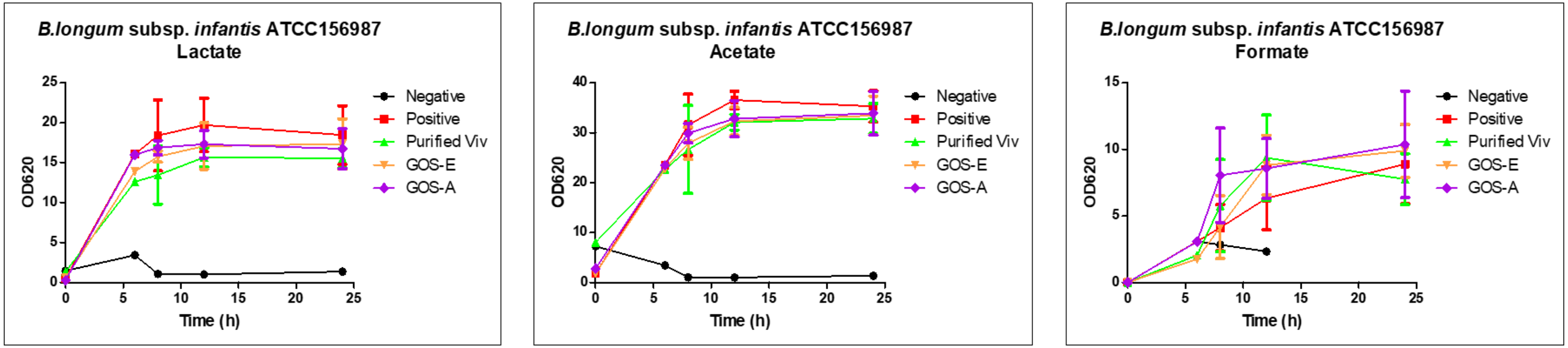

Supplementary Figure S2.f SCFA production resulting from *B.longum* subsp. *infantis* ATCC156987 growth on novel purified GOS preparations. Negative: no sugar, Positive: lactose, Purified Viv: Pure Viv, GOS-E: purified GOS obtained by enzymatic reaction employing BgaE, GOS-A: purified GOS obtain by enzymatic reaction employing BgaA.

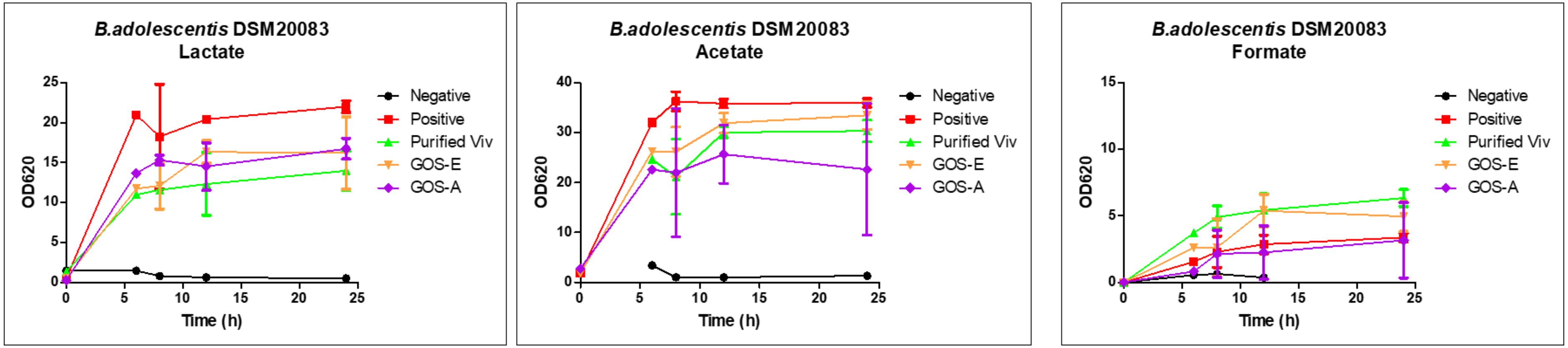

Supplementary Figure S2.g SCFA production resulting from *B.adolescentis* DSM20083 growth on novel purified GOS preparations. Negative: no sugar, Positive: lactose, Purified Viv: Pure Viv, GOS-E: purified GOS obtained by enzymatic reaction employing BgaE, GOS-A: purified GOS obtain by enzymatic reaction employing BgaA.

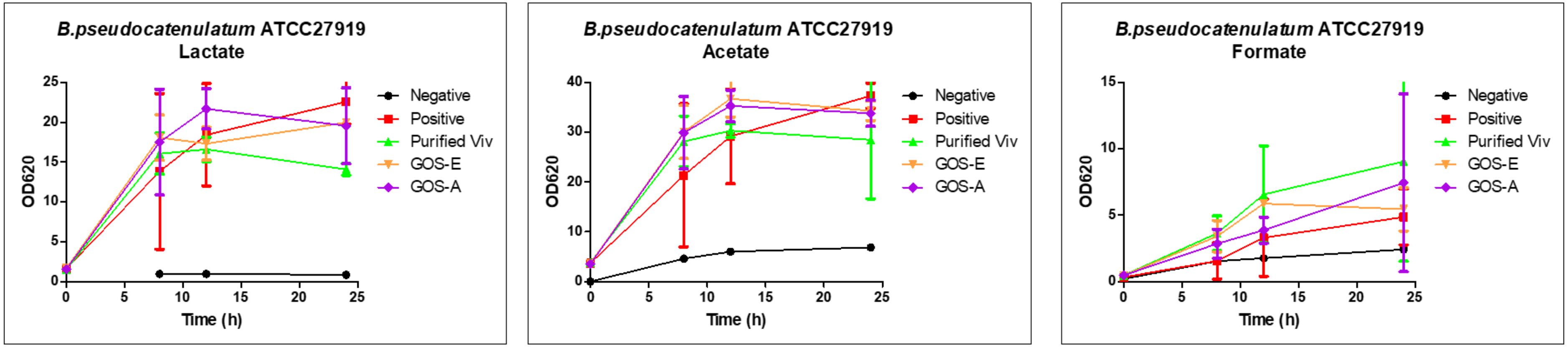

Supplementary Figure S2.h SCFA production resulting from *B.pseudocatenulatum* ATCC27919 growth on novel purified GOS preparations. Negative: no sugar, Positive: lactose, Purified Viv: Pure Viv, GOS-E: purified GOS obtained by enzymatic reaction employing BgaE, GOS-A: purified GOS obtain by enzymatic reaction employing BgaA.

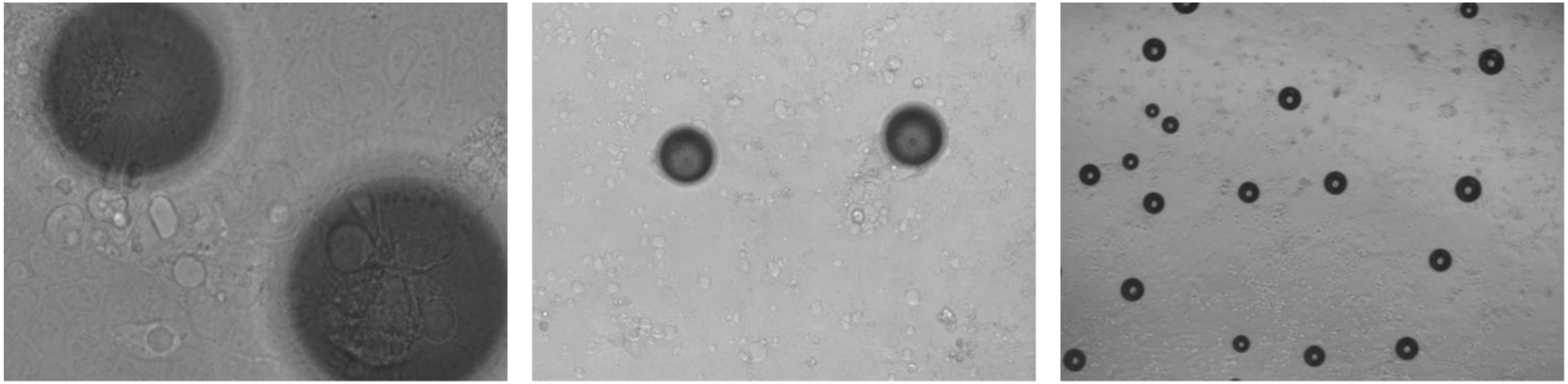

Supplementary Figure S3. C2BBe1 cell line infected with *Salmonella typhimurium* 4/74, micro bubble formation.
